# Evolutionary Analysis of Human Immunodeficiency Virus Type 1 Therapies Based on Conditionally Replicating Vectors

**DOI:** 10.1371/journal.pcbi.1002744

**Published:** 2012-10-25

**Authors:** Ruian Ke, James O. Lloyd-Smith

**Affiliations:** 1 Department of Ecology and Evolutionary Biology, University of California, Los Angeles, Los Angeles, California, United States of America; 2 Fogarty International Center, National Institutes of Health, Bethesda, Maryland, United States of America; Utrecht University, Netherlands

## Abstract

Efforts to reduce the viral load of human immunodeficiency virus type 1 (HIV-1) during long-term treatment are challenged by the evolution of anti-viral resistance mutants. Recent studies have shown that gene therapy approaches based on conditionally replicating vectors (CRVs) could have many advantages over anti-viral drugs and other approaches to therapy, potentially including the ability to circumvent the problem of evolved resistance. However, research to date has not explored the evolutionary consequences of long-term treatment of HIV-1 infections with conditionally replicating vectors. In this study, we analyze a computational model of the within-host co-evolutionary dynamics of HIV-1 and conditionally replicating vectors, using the recently proposed ‘therapeutic interfering particle’ as an example. The model keeps track of the stochastic process of viral mutation, and the deterministic population dynamics of T cells as well as different strains of CRV and HIV-1 particles. We show that early in the co-infection, mutant HIV-1 genotypes that escape suppression by CRV therapy appear; this is similar to the dynamics observed in drug treatments and other gene therapies. In contrast to other treatments, however, the CRV population is able to evolve and catch up with the dominant HIV-1 escape mutant and persist long-term in most cases. On evolutionary grounds, gene therapies based on CRVs appear to be a promising tool for long-term treatment of HIV-1. Our model allows us to propose design principles to optimize the efficacy of this class of gene therapies. In addition, because of the analogy between CRVs and naturally-occurring defective interfering particles, our results also shed light on the co-evolutionary dynamics of wild-type viruses and their defective interfering particles during natural infections.

## Author Summary

A long-standing challenge in efforts to control human immunodeficiency virus type 1 (HIV-1) is the rapid evolution of the virus. Any effective therapy quickly gives rise to so-called escape mutants of the virus, potentially resulting in treatment failure. A distinct class of gene therapy based on conditionally replicating vectors has been suggested to have potential to circumvent the problem of viral evolutionary escape. A conditionally replicating vector cannot replicate on its own, but when it coinfects the same cell with HIV-1, it is packaged into a virion-like particle and can be transmitted from cell to cell. Importantly, these vectors replicate using the same machinery that HIV-1 uses, and so they mutate at the same rate. This opens the possibility that conditionally replicating vectors could ‘keep up’ with HIV-1 evolution and prevent HIV-1 escape. In this study, we present mathematical analyses of the co-evolutionary dynamics of HIV-1 and conditionally replicating vectors within a patient. Our results show that with proper genetic design, conditionally replicating vectors can keep pace with HIV-1 evolution, leading to persistent reduction in HIV-1 viral loads. Therefore, this class of gene therapies shows potential for ‘evolution-proof’ control of HIV-1, and merits further investigation in laboratory trials.

## Introduction

The HIV-1 pandemic has been a major challenge in global public health for decades, and continues to impose crippling burdens of morbidity and mortality worldwide. While recent years have brought major breakthroughs in identifying the protective effect of male circumcision [1], [2], [3] and the transmission-blocking potential of early antiretroviral (ARV) drug therapy [4], the scalability and sustainability of these strategies remains in doubt. ARV therapy can reduce viral loads to undetectable levels, but affected populations must be reached, continuous treatment (and hence investment) is required, long-term ARV use can result in side effects, and there is an ever-present risk that the virus will evolve drug resistance [5], [6], [7]. Meanwhile efforts to develop a protective vaccine, the conventional tool for broad-scale disease prevention, have been unsuccessful so far [8]. As a result, alternative strategies are being investigated, including gene therapy approaches. Gene therapies offer many advantages compared with pharmaceutical drugs, such as low economic cost, ease of administration, and potential to reduce HIV-1 viral loads by sustained interference with the viral life cycle (reviewed in [9], [10]).

All methods of HIV-1 treatment, and many methods of prevention, are challenged by the extremely rapid evolution of the HIV-1 genome. The error-prone reverse transcription of HIV-1 generates a ‘viral swarm’ of HIV-1 genotypes within an individual host. This population of HIV-1 virions is under continual selection, from immune system effectors and medical treatments, resulting in the rapid generation of immune escape variants and drug-resistant mutants. Such resistant mutants are frequently observed in patients under long-term drug treatment and gene therapy [11], [12], [13], [14], [15].

One family of gene therapy approaches offers the intriguing possibility of overcoming the challenges caused by rapid HIV-1 evolution. Conditionally replicating vectors (CRVs) have been discussed as a gene therapy strategy to combat HIV-1 for two decades [16], [17], [18]. Like other approaches to antiviral gene therapy, CRVs are capable of delivering genetic elements into the host cell's nuclear genome to block HIV-1 replication through inhibition and competition [16], [19], [20], [21], [22]. Many proposals for CRVs center on genetic modification of HIV-1 or other lentiviruses, including the addition of viral inhibitory machineries and the deletion of essential genes for viral replication or packaging. The defining characteristic of CRVs is that they can create new virions, and hence transmit from cell to cell, by complementation with wild-type HIV-1; because of this trait, CRVs are also known as mobilization-competent vectors [19], [23]. Because of the clear analogy to naturally-occurring defective interfering particles [24], [25], [26], [27], [28], [29], the term therapeutic interfering particle (TIP) has also been proposed [30].

In contrast to other therapeutic approaches, the CRV approach offers a unique opportunity to overcome the problem of HIV-1 evolution [19], [30]. Because CRVs replicate using the exact same machinery as HIV-1, they have potential to evolve as rapidly as HIV-1. Consequently, it is possible that CRVs can hold their ground in a co-evolutionary arms race, and continuously interfere with HIV-1 replication by generating mutants that match HIV-1 escape mutants. This phenomenon has been reported previously for defective interfering particles [31], [32], [33], [34]. Recently, an experimental study has shown that a CRV expressing small non-coding RNAs that targeted the long terminal repeat (LTR) of HIV-1 was able to suppress HIV-1 viral production without viral escape for one month [19]. A clinical trial has shown that a CRV can be maintained stably within patients, and the long-term presence of the vector does not induce adverse clinical effects [21]. These results highlight the unique promise of CRVs as a strategy for HIV-1 intervention. However, the co-evolutionary dynamics of HIV-1 and CRVs have not been investigated in detail, and the conditions that would allow CRVs to persistently suppress HIV-1 abundance are not known. Important questions have been raised about the conditions that would allow escape mutants of HIV-1 to arise, and the outcomes of co-evolution between the HIV-1 and CRV genomes [9], [11], [16], [19], [21], [22], [30], [35]. In addition, it is possible that CRVs could select for faster replicating HIV-1 strains, thereby potentially causing more severe disease in those susceptible individuals who are not protected [36].

Here we provide the first investigation of the co-evolutionary dynamics associated with the use of a CRV gene therapy against HIV-1. We use a mathematical modeling approach, building upon the best-developed model framework for the within-host dynamics of a CRV therapy against HIV-1 [30], [37]. Thus our analysis focuses on a particular proposed therapy, the therapeutic interfering particle or TIP, but this case study enables us to address general questions about the evolutionary robustness of CRV therapies against HIV-1. Mathematical modeling is well established as a tool for elucidating the mechanisms underlying viral dynamics and evolution [38], [39], [40], and has contributed greatly to our understanding of the dynamics of HIV-1 and the immune system [41], [42], [43], as well as the mechanisms by which antiviral drugs act on the HIV-1 population within hosts [43], [44], [45]. Models have been also used for assessing the potential properties and refining design principles of those proposed interventions, such as novel gene therapies, for which *in vivo* data are currently lacking. Recently, several modeling studies have investigated the properties of TIPs. Weinberger *et al.* modeled the *in vivo* dynamics of HIV-1 and TIPs, and showed that TIPs could reduce the HIV-1 viral load by orders of magnitude to a level comparable to that caused by highly active ARV treatment [37]. In another study, Metzger *et al.* extended the previous model to study the dynamics of HIV-1 and TIP at three levels (intracellular, within-host and population) to understand the population-level consequences of TIP intervention [30]. That study made qualitative arguments about the likely direction of selection acting on TIP and HIV-1 at different scales, and predicted that competing selection pressures across scales would lead TIP therapies to be evolutionarily robust. However the co-evolutionary dynamics are not considered explicitly in either study, so these hypotheses remain untested.

We study the co-evolutionary dynamics of TIPs and HIV-1 in the peripheral blood within a host with the aim of establishing design principles for the class of CRV gene therapies and addressing safety concerns associated with them. Our aims are to evaluate the long-term persistence and efficacy of TIPs, to clarify the likely selective pressures on HIV-1 arising from the presence of TIPs, and to test whether HIV-1 can evolve resistant mutants that escape TIP inhibition. By constructing and analyzing mathematical models, we show that the dynamics of HIV-1 and TIP follow a characteristic three-phase pattern. The first two phases reflect temporary efficacy of the therapy followed by evolutionary escape of HIV-1, as observed in studies of antiviral drugs as well as other gene therapy approaches. The TIP is strikingly different, though, because under most conditions, it is able to catch up to HIV-1 evolution and continue to exert its therapeutic effects even after the HIV-1 escape mutant has arisen. We hypothesize that this third phase of sustained suppression of HIV-1 may be a general feature of well-designed CRV therapies, as indicated by preliminary empirical findings [19]. By performing sensitivity analysis, we find that the qualitative behavior of the system is robust to differing assumptions about the detailed interactions between HIV-1 and TIP, supporting this notion. We conclude by using our model to propose possible design criteria to enhance the efficacy and robustness of this class of CRVs in the context of HIV-1 evolution.

### Case study and model overview

The TIP is a proposal for a genetically engineered CRV to combat HIV-1 [30], [37]. It would have a genomic structure that is closely similar to HIV-1, but lacking all the structural and envelope genes required for replication and packaging. Upon infection, the TIP integrates its genome into the host cell, but it can only replicate when the cell is further infected by HIV-1 such that the gene products required for viral replication and packaging are available. The TIP would be designed so that its genome is synthesized at a much higher rate than that of HIV-1, resulting in higher production of TIP virions from a dually infected cell. In addition, the TIP can be genetically modified to encode inhibitory agents, such as RNAi [46], [47] and ribozyme nuclease [48], to inhibit HIV-1 production by blocking its life cycle. As explored in earlier modeling studies [30], [37], the efficacy of TIPs is driven by dynamics at several scales. At the cellular scale, resource competition between TIPs and HIV-1 and the direct inhibition of HIV-1 production result in reduced production of new HIV-1 virions [30]. At the scale of within-host dynamics, infection with TIP results in a lower overall HIV-1 viral load (see Refs. 30,37 for detailed analyses of the dynamics between HIV-1 and the TIP without considering evolution). Because TIPs are packaged in HIV-1 structural elements, it has hypothesized that they could transmit between hosts using the same routes as HIV-1, but we do not consider this phenomenon here.

**Figure 1:**
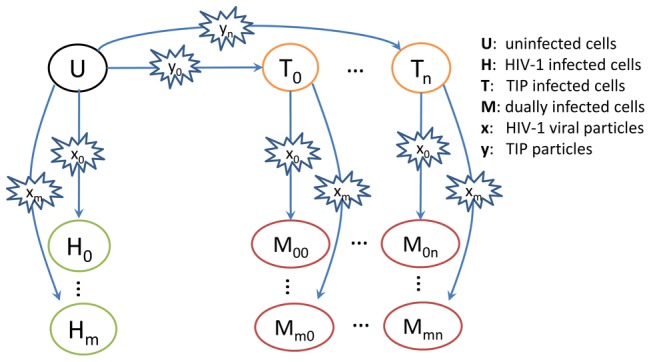
A schematic of the multi-strain HIV-1 and TIP system at the within-host level. At the within-host level, the uninfected CD4^+^ T cell (‘U’) can be infected by one of *m* HIV-1 strains or one of *n* TIP strains, becoming an HIV-1 infected cell (‘H_i_’, *i = 0,1,..m*) or a TIP infected cell (‘T_j_’, *j = 0,1,…n*), respectively. A TIP infected cell can be further infected by one of *m* HIV-1 strains, resulting in m*n different types of dually infected T cells (‘M_ij_’).

We developed a cross-scale model of the co-evolutionary dynamics of HIV-1 and TIP, based on the biological framework proposed previously by Metzger *et al.* [30] (Fig. 1). The model keeps track of the competition of multiple strains of HIV-1 and TIP at both the cellular level and the host level. At the host level, infection of susceptible CD4^+^ T cells (*U*) by the *i^th^* HIV-1 viral strain (denoted as *x_i_*, *i* = *1…m*) or by the *j^th^* TIP strain (denoted as *y_j_*, *j* = *1…n*) leads to HIV-1 infected cells (*H_i_*) or TIP infected cells (*T_j_*), respectively. T cells infected by *y_j_* (*T_j_*) can be further infected by *x_i_*, becoming dually infected cells (*M_ij_*). New HIV-1 viral particles are produced from both HIV-1 infected cells and dually infected cells, whereas new TIP particles are produced only from dually infected cells, since the materials needed for replication and packaging are lacking in TIP infected cells (*T_j_*).

At the cellular level, both the HIV-1 genome and the TIP genome are transcribed to genomic RNAs (gRNAs) in dually infected cells. We follow the assumption of Metzger *et al.* [30] that dimerization and encapsidation of two copies of genomic RNAs result in three types of proviruses: HIV-1 homozygotes, TIP homozygotes, and heterozygotes with one copy of HIV-1 gRNA and one copy of TIP gRNA. Heterozygotes are not viable to infect other cells owing to the difference in length between the gRNAs of HIV-1 and TIP [30]. Therefore, TIP production is proposed to reduce HIV-1 yield in a cell through pairing with HIV-1 genomes, i.e. a form of resource competition. To further reduce HIV-1 production, the TIP can be engineered to encode an inhibitory factor, such as an RNAi, which blocks the process of HIV-1 genome production and virion formation. Broadly speaking, for the class of gene therapies based on CRVs, the number of HIV-1 virions produced by the dually infected cell can be reduced by two factors: competition with CRV genomes for resources, and direct inhibition by inhibitory factors encoded by CRV genomes. The intracellular model constructed for the TIP is a special case for the general interactions of competition and inhibition between CRVs and HIV-1 genomes.

To model the molecular evolution of HIV-1 and TIP, we assume that the cellular-scale phenotypes of HIV-1 and TIP are determined by the genomes of HIV-1 and TIP and the interactions between them. The genotype-phenotype mapping is determined using a parsimonious approach recently introduced for co-evolutionary models of viruses and the immune system [41], [49]. Each HIV-1 or TIP strain is represented by a digital sequence, reflecting the genetic elements involved in controlling the traits of interest, and trait values arising from interactions between HIV-1 and TIP are determined by a string-matching algorithm (see Methods).

Building on the model of Metzger *et al.* [30], we consider three critical phenotypic parameters (*D*, *P*, and *A*) in the cellular model. Parameter *D* is the *fraction of inhibition/upregulation* of HIV-1 gRNA production, and it models the degree of inhibition exerted by the TIP-encoded repressor on the production of HIV-1 gRNA in a dually infected cell. Mutations in the gene encoding the repressor in the TIP genome, or in the region where the repressor binds in the HIV-1 genome, can change the value of *D* [30], [37]. We allow the value of *D* to vary over a range from the situation where HIV-1 production is completely blocked by the inhibitory factor (*D* = 1) to the situation where the inhibitory factor has evolved to become an activator of HIV-1 production (*D*<0). Note that since TIP replication and packaging require materials produced by the HIV-1 genome, inhibition of HIV-1 production affects TIP production in a similar way (see Methods). Parameter *P* is the *production ratio* of TIP gRNA over HIV-1 gRNA in a dually infected cell. This is the relative rate of TIP genome replication in a dually infected cell, compared to the rate of wild-type HIV-1 genome replication. The value of parameter *P* will be influenced by the interaction between TIP gRNA and the HIV-1 proteins Rev and Tat, as well as TIP's genome length and manipulations to its splice sites [30], [37]. Accordingly we assume that the value of *P* is jointly controlled by the two genomes. Parameter *A* is the *replication coefficient*, which characterizes the genome replication rate of a HIV-1 strain in a cell infected only with HIV-1, relative to the genome replication rate of the wild-type HIV-1 strain. This factor is a simple phenomenological representation of the evolutionary constraints on the HIV-1 genome, since there may be a fitness cost to mutating away from the wild-type genotype that prevails in the absence of TIPs. Only mutations in the HIV-1 genome can change the value of *A*.

We implemented these intracellular and within-host mechanisms using a hybrid deterministic-stochastic framework, following techniques developed elsewhere [41], [50]. The dynamics of existing strains of HIV-1 and TIP are modeled by ordinary differential equations, while mutation events are modeled stochastically.

## Results

### Static fitness landscapes

To gain insight into the competition between different strains of HIV-1 and TIP, we started with the two simplest evolutionary scenarios: the ‘HIV-1 mutant’ model and the ‘TIP mutant’ model (see Eqn.S7 and S8 in Text S1). Each of these models considers only three strains: wild-type or ‘resident’ strains of HIV-1 and TIP, and a mutant strain of either HIV-1 or TIP. We assumed that infection with a mutant strain results in different *D*, *P* and *A* values compared to the wild-type strain (see Methods section for details). To reveal the fitness landscape of the mutant against a fixed resident viral population in a host, we performed invasion analysis, in which the effective reproduction number, R_eff_, of the mutant is calculated when it is introduced into a system where both the resident HIV-1 and the resident TIP are at equilibrium [51]. R_eff_ is a measure of the relative fitness of the mutant compared to the resident strain. When R_eff_>1, the mutant can invade the system and replace the resident strain, and when R_eff_<1, the mutant is unable to invade and dies out. We used R_eff,H_ and R_eff,T_ to denote the effective reproduction numbers of the mutant HIV-1 in the ‘HIV-1 mutant’ model and the mutant TIP in the ‘TIP mutant’ model, respectively. Analytic expressions for both quantities are shown in equations (5) and (6) in the Methods section.

**Figure 2:**
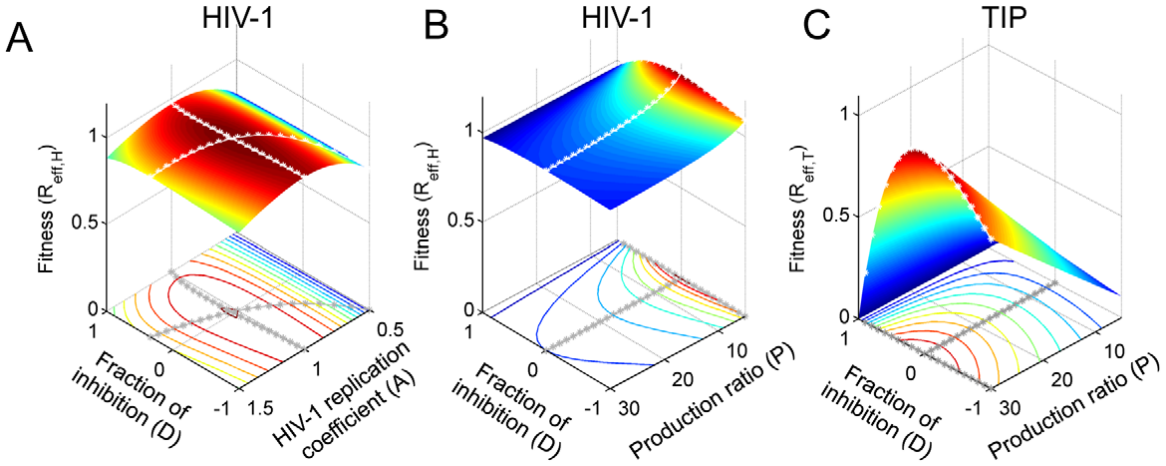
The fitness landscapes for HIV-1 and TIP mutants in the 3-strain ‘mutant invasion’ models. The fitness was calculated as the effective reproduction number of the mutant when it is introduced into a host with the wild-type HIV-1 and the wild-type TIP at equilibrium. (**A**) The invasion fitness of HIV-1 mutants (R_eff,H_) with different values of the HIV-1 replication coefficient, *A*, and the fraction of inhibition, *D*. Positive values of *D* correspond to inhibition of HIV-1 gRNA production, while negative values correspond to upregulation. (**B**) The invasion fitness of HIV-1 mutants (R_eff,H_) with different values of the fraction of inhibition, *D*, and production ratio, *P*. (**C**) The invasion fitness of TIP mutants (R_eff,T_) with different values of the fraction of inhibition, *D*, and production ratio, *P*. In all three plots, two parameter values were varied while keeping the third one fixed; baseline values are *P* = 30, *D* = 0, *A* = 1. Lines of white stars in the plots show the maximal fitness for one parameter while other parameter values are fixed.

We first analyzed the selection pressure on HIV-1 replication, i.e. how the fitness of mutant HIV-1 strains varies as a function of the replication coefficient *A*. Several lines of evidence show that increases in viral gRNA replication rate beyond the wild-type rate have negative impacts on overall fitness, since the death rate of the infected T cell increases dramatically as viral gRNAs and virus-encoded proteins accumulate inside the cell [52], [53]. In the model, we follow previous studies [54], [55] in assuming that the wild-type replication rate (*A = 1*) has evolved to be optimal in the absence of TIP, and modeling the dependence of the death rate of the HIV-1 infected T cell on the production rate of HIV-1 gRNA with a concave-up function (*Ω*(*A*)) (see Methods). In a dually infected host, we found that the optimal replication rate at which HIV-1 attains maximal fitness in the presence of TIP is very close to the wild-type value (Fig. 2A). This is because the majority HIV-1 virions are produced from singly infected cells, and thus the overall fitness of HIV-1 within hosts is more affected by changes of HIV-1 production in singly infected cells than in dually infected cells. Therefore, HIV-1 strains with notably higher replication rates, which potentially could be more virulent, are unlikely to be selected and transmitted to other hosts.

The parameters *D* and *P* are determined by the genomes of both HIV-1 and TIP, so we considered their impact on the fitness of both types of virus. For the fraction of inhibition (*D*), we allowed it to vary between −1 and 1 to consider both upregulation and inhibition of HIV-1 production by TIP-encoded elements. The optimal value of *D* for HIV-1 varies with the rate of HIV-1 gRNA replication (Fig. 2A). However since we have shown that the HIV-1 gRNA replication rate will stay near the wild-type level of *A* = 1 in the presence of TIP, the optimal value of *D* for HIV-1 is 0 (Fig. 2A). Considering the fitness of TIP mutants, we found that the optimal value of *D* for TIP is also 0 when *A* = 1 (Fig. 2C). This suggests that selection will lead the inhibitory factor encoded by TIP to become non-functional over time.

The parameter *P* characterizes the relative rate of TIP gRNA production in a dually infected cell. In the model, we allowed *P* to vary from 0 to 30 in line with arguments presented by Metzger *et al.* [30]. Higher *P* values cause dually infected cells to produce more TIP virions, while at the same time wasting HIV-1 resources. Consequently, selection on HIV-1 favors low *P* values (Fig. 2B), while selection on TIP favors high *P* values (Fig. 2C), as argued previously [30].

To ensure robustness of these results, we further tested two major assumptions made in this analysis. We first tested the sensitivity of the optimal *A* and *D* values for HIV-1 to the assumed relationship between the HIV-1 replication rate and the death rate of infected T cells (Fig. S1A). If the death rate of infected T cells is a concave-up function of HIV-1 replication rate, then fitness decreases as the replication rate increases beyond the wild-type level. When the curvature of this relationship is at least moderate as assumed in our main analyses (*α* = 1 in the function *Ω*), then the optimal values of *A* and *D* remain close to 1 and 0 (the wild-type values), respectively (Fig. S1B). As the curvature becomes weaker, i.e. the stiffness parameter *α* becomes smaller, the optimal values of *A* and *D* become larger. If the death rate of HIV-1 infected cells is a linear function of HIV-1 replication (*Ω*(x) = 0.7*x; the black line in Fig. S1A), the total number of virions produced in a cell would stay the same irrespective of variations in the HIV-1 genomic production rate, i.e. variations in HIV-1 replication rate do not change its fitness. It can then be concluded from Eqns. (1) and (5) in the Methods section that changes in the values of *A* and *D* do not affect the value of R_eff,H_, and therefore, the presence of CRVs does not select for HIV-1 variants with higher replication rate in this scenario. Second, we explored the possibility of mutation in the Dimerization Initiation Signal (DIS) region of the HIV-1 and TIP genome, which changes the rates of dimerization of different single-stranded genomic RNAs, and thereby changes the distribution of diploid genomes. Intuitively, a lower rate of heterodimer formation would result in higher production of both HIV-1 and TIP virions in dually infected cells, and thus would raise HIV-1 and TIP fitness. However, this benefit may be balanced by fitness costs arising from mutating the DIS region. Numerous experimental studies, as well as the conservation of the DIS sequence in wild-type HIV-1, indicate that mutations in the DIS region lead to reductions in viral fitness [56], [57], [58]. By incorporating conservative assumptions about this reduction in viral fitness into our invasibility model, we found that mutations in the DIS region are not likely to invade for either HIV-1 or TIP (see Text S1).

Taken together, our analyses of mutant invasibility models made the following predictions for HIV-1 and TIP co-evolution (summarized in Table 1): 1) the HIV-1 replication rate will stay at approximately the wild-type level; 2) the TIP-encoded inhibitory factor, if any, will evolve toward a non-functional state (*D* = 0); and 3) there is a conflict between selection pressures on HIV-1 and TIP regarding the *production ratio*, *P*, i.e. selection on HIV-1 favors low *P* values, but selection on TIP favors high *P* values. This final conflict sets the stage for a co-evolutionary arms race.

**Table 1:** Optimal parameter values for HIV-1 fitness and TIP fitness, respectively, in the mutant invasibility models.

Parameters	Value for the wild-type strains	HIV-1 optimum	TIP optimum
P	30	0 (min)	30 (max)
D	0	0	0
A	1	1	N/A

### Co-evolutionary dynamics

**Figure 3:**
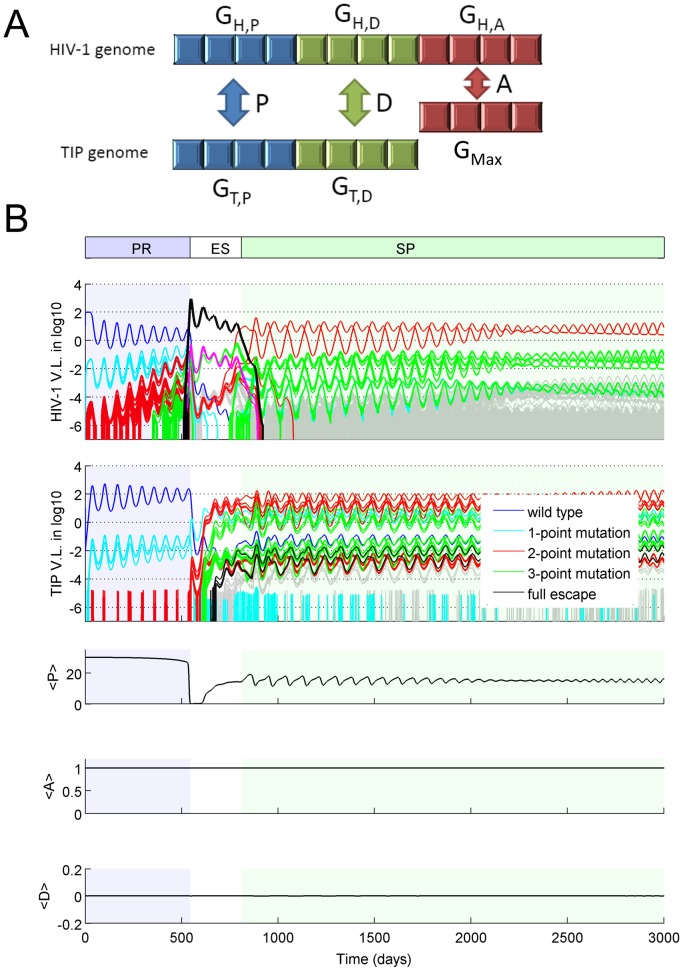
Co-evolutionary dynamics of HIV-1 and TIP. (**A**) A schematic for the genotype-phenotype mapping in a dually infected T cell. The values of parameters *P* and *D* are calculated by comparing the appropriate genome regions of HIV-1 (G_H,P_ and G_H,D_, respectively) with the corresponding genome regions of TIP (G_T,P_ and G_T,D_, respectively). The parameter *A* is calculated by comparing the appropriate genome region of HIV-1 (G_H,A_) to a genome sequence that allows HIV-1 gRNA to replicate at the maximal rate (G_Max_). (**B**) A typical realization of the co-evolutionary dynamics of HIV-1 and TIP. The dynamics can be broken into three phases depending on the dominant HIV-1 strain: the ‘preliminary’ phase (PR) dominated by the wild-type HIV-1 strain, the ‘escape’ phase (ES) dominated by the full-escape mutant, and the ‘set-point’ phase (SP) dominated by the match-escape pairs described in the text. The top two panels show the viral loads per µL (in log scale) for each HIV-1 strain and each TIP strain, respectively. The curve for each strain is color-coded according to their genetic distance to the wild-type strain: Blue, cyan, red, green, black denote the wild-type and the mutants with one-, two-, three-point mutations and the full-escape mutants, respectively. The bottom three panels show the mean values of parameter *P*, *A* and *D* across the population of dually infected cells at each point in time. For these simulations, the genome was modeled as a binary string, and the length of the genome region corresponding to each parameter was 4 sites.

To explore the co-evolutionary dynamics arising from the conflict in selection pressure on *P*, and to test the predictions of the invasibility analysis, we constructed a multi-strain deterministic-stochastic hybrid model. In the model, each HIV-1 or TIP strain has a unique digital sequence, representing the relevant part of the genome of HIV-1 or TIP (Fig. 3A), and the genotype-phenotype mapping is determined using a recently developed string-matching approach to modeling co-evolution [41], [49]. The parameters *P* and *D* (phenotypes) are determined by the degree of matching between the digital sequences of the HIV-1 and TIP (genotypes) infecting a cell (G_H,P_ and G_H,D_ in the HIV-1 genome, and G_T,P_ and G_T,D_ in the TIP genome, shown in Fig. 3A and explained in the Model Overview section); the parameter *A* is determined by the match between the HIV-1 genotype and a genotype that allows HIV-1 to replicate at a maximal rate (G_H,A_ in HIV-1 genome and G_Max_ in TIP genome, Fig. 3A). Therefore, mutations in the genomes lead to altered intracellular parameters, which in turn lead to changes in the fitness of the mutant virions (see Methods section for details).

Simulation results confirmed the predictions from the invasion analysis with respect to parameters *A* and *D*, which stayed close to 1 and 0, respectively (Fig. 3B). The dynamics for parameter *P* are more complex as a result of the opposing selection pressures on HIV-1 and TIP. Considering the broader dynamics of co-evolution, we found that the system exhibited a robust pattern of three distinct phases, namely the ‘preliminary’ phase (PR), the ‘escape’ phase (ES) and the ‘set-point’ phase (SP). Below, we first describe the three phases in a typical realization of the model (shown in Fig. 3B), then we describe the sensitivity analysis that tested the robustness of the results to the assumptions made.

The ‘preliminary’ (PR) phase is characterized by the dominance of both the wild-type HIV-1 and the wild-type TIP in the viral population. During this phase, the average *P* value (mean across all extant infected T cells, denoted *<P>*) was high (close to the maximum value of 30) and the HIV-1 viral load was reduced to a low level, as a result of the high efficacy of the genetically engineered wild-type TIP strain. However, mutant HIV-1 strains were generated rapidly within the host. Those mutant HIV-1 particles that lead to a lower *P* value when co-infecting a T cell with the wild-type TIP (i.e. those with mutations in the G_H,P_ region in Fig. 3A) possess selective advantages over the wild-type, and thus rose in frequency within the host. Eventually the ‘full escape’ mutant (highlighted as heavy black line in Fig. 3B), whose genomic sequence in the G_H,P_ region is completely different from the G_T,P_ region of the wild-type TIP, was generated. The level of the full-escape HIV-1 mutant increased exponentially after emergence, since it is completely released from suppression by TIP. Consequently <*P*> decreased rapidly at the end of the PR phase. In contrast to the rising genetic diversity of the HIV-1 population, the TIP mutants with one point mutation in the G_T,P_ region (i.e. the one-point TIP mutants) were generated quickly but remained at a relatively low level during the PR phase. Two-point TIP mutants arose repeatedly throughout the PR phase, but were cleared rapidly from the system each time. TIP evolutionary dynamics differed from HIV-1 in the PR phase because TIP mutants were not selectively advantageous, due to their low fitness when the HIV-1 population was dominated by the wild-type strain.

The ‘escape’ (ES) phase is the time period during which the full-escape HIV-1 mutant dominates the population. The HIV-1 viral load increased to a high level at the beginning of this phase, as a result of HIV-1 escape from suppression by TIP. The average *P* value in the host (*<P>*) dropped almost to 0. However, because the majority of the HIV-1 infected T cells were infected with the full-escape mutant, the extant TIP mutants (the 1-point and 2-point TIP mutants) that partially match the full-escape HIV-1 genome possessed selective advantages over the wild-type TIP at the beginning of the ‘ES’ phase, and thus increased in frequency. These mutants gave rise to 3-point TIP mutants, which possessed further advantage in *P*. Once the 3-point TIP mutants became the dominant strains, the HIV-1 full-escape mutant was replaced by another strain that has the lowest match with the three-point TIP mutant in their representative genome sequences for parameter *P*. This marked the end of the ES phase; diverse HIV-1 and TIP strains were present in relatively high abundance, resulting in intermediate values of *<P>* fluctuating around 15.

The system then entered the ‘set-point’ (SP) phase where HIV-1 and TIP settled into a stable coexistence, in which the abundance of each HIV-1 or TIP strain oscillated around a fixed point, and the populations of HIV-1 and TIP were each dominated by two strains (the uppermost red lines in Fig. 3B, with mean long-term abundances at least 10-fold higher than any other strains). By examining the genome sequences of these strains in the regions determining *P* values, we found that they consist of two matched pairs that are opposite to each other, i.e. each dominant HIV-1 strain has a perfectly matched TIP strain, and the two such pairs are complete mismatches of each other. Therefore, the oscillatory dynamics between the two dominant strains of HIV-1 and TIP can be understood as a ‘match-escape’ cycle: a TIP strain increases in abundance by matching the dominant HIV-1 strain, the HIV-1 strain that escapes the control of this TIP is selected, leading to selection for the other TIP strain, and so on. The oscillatory dynamics resulted in average *P* values oscillating near an intermediate value of around 15. The set-point viral load of HIV-1 is at 15 virions*/*µ*L* on average; in contrast, this model predicts a mean HIV-1 viral load of 100 virions*/*µ*L* in the absence of TIP. As emphasized in earlier work [30], [37], this sustained suppression of HIV-1 is possible because of the high abundance of TIP particles, which leads to a majority of T cells being TIP-infected. As a result, a large proportion of T cells infected with HIV-1 at set-point are co-infected with TIP, and HIV-1 replication is robustly suppressed.

**Figure 4:**
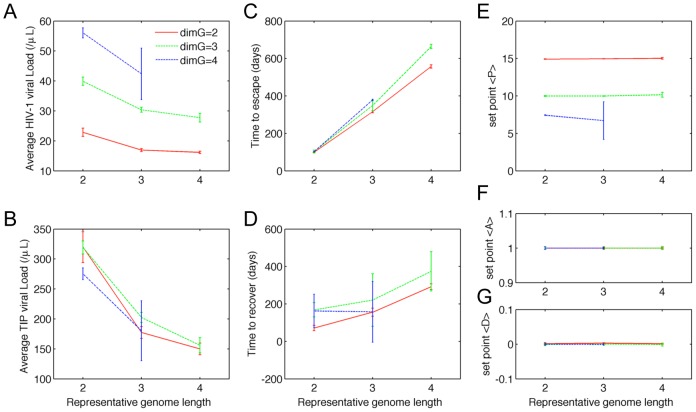
The co-evolutionary dynamics are qualitatively robust to variation in genome size and dimension. We tested model sensitivity to the dimension of genome space (i.e. the number of values each genome site could have; denoted dimG) and the genome length (i.e. the number of sites corresponding to each parameter. The co-evolutionary dynamics of HIV-1 and TIP were characterized by 7 attributes: the mean HIV-1 and TIP viral loads (panel (A) and (B)) over the whole simulation (3000 days), the lengths of the PR phase and the ES phase (panel (C) and (D)), and the time-average mean values of *P*, *A* and *D* of the population of dually infected cells (panel (E), (F) and (G)) during the ‘set-point’ phase. Data points and error bars correspond to the mean and standard deviation of 100 realizations of the model for those parameter values.

To test whether the results above are robust to changes in the genomic structure, we performed simulations for models assuming higher genome dimensions instead of binary sequences, and different lengths of genome regions corresponding to the phenotypes of interest. For all simulations performed, TIP evolution was able to catch up with HIV-1 after the emergence of the full-escape mutant, leading to establishment of the set-point phase. We quantified the average HIV-1 and TIP viral loads, the average values of *P*, *D* and *A*, and the duration of each phase of the dynamics, and found that the qualitative behavior of the system is robust to changes in genome structure (Fig. 4). There were some quantitative changes in the dynamics, which accord with our intuitive understanding of the system. Longer representative genome lengths led to lower HIV-1 viral loads (Fig. 4A), since it took longer for an HIV-1 escape mutant to arise in those simulations (Fig. 4B). In contrast, higher genome dimensions led to higher HIV-1 viral loads. This is because, during the set-point phase, more HIV-1 mutant strains were available to escape the dominant TIP, however, only one TIP strain was able to match the dominant HIV-1 strain. In essence, the HIV-1 population had a larger genotype space in which to escape suppression by TIP, leading to a lower set-point <P> (Fig. 4E).

The fraction of dually infected cells is an important parameter determining the viability of TIP, as well as other evolutionary outcomes for HIV-1 [59]. In peripheral blood of untreated HIV-1 patients, the frequencies of multiple infection among all infected CD4^+^ T cells are 2.6% and 7.0% for acute and chronic infection, respectively [60]. Substituting our model parameters into a model for HIV-1 superinfection following the approach developed in a recent study [59], we obtain a prediction that 2.6% of all infected cells will be multiply infected, indicating that our assumed superinfection rate maps onto the lower range of observed values (Text S1). In our simulations, the frequency of T cells dually infected by HIV-1 and TIP among all infected cells is around 0.4%, which is much lower than the predicted value. This is because the majority of dually infected cells would be infected by two TIPs (which is not considered in our model), as a result of effective TIP suppression of HIV-1 viral loads. To explore the potential impact of higher frequencies of dual infection, we extended the model to include the superinfection of HIV-1 infected cells by TIP, and further tested the sensitivity of our results to increases in the superinfection rate. Importantly, the three-phase co-evolutionary dynamics are robust to changes of superinfection rate and the frequency of dually infected cells (Text S1). Increasing the rate of the superinfection rate leads to higher TIP fitness and therefore a lower minimum value of *P* (*P*_threshold_) required for TIP invasion (compare Eqn.(4) in the Method section and Eqn.(S15) in Text S1), suggesting that TIP performs better when the superinfection rate increases. However, increasing the rate of superinfection shortens the time required to generate the HIV-1 full-escape mutant, because higher frequency of dually infected cells leads to higher selection pressure on HIV-1 to escape, but the TIP always catches up and the system approaches the same set-point as in our main analysis.

### Alternative genotype-phenotype maps

**Figure 5:**
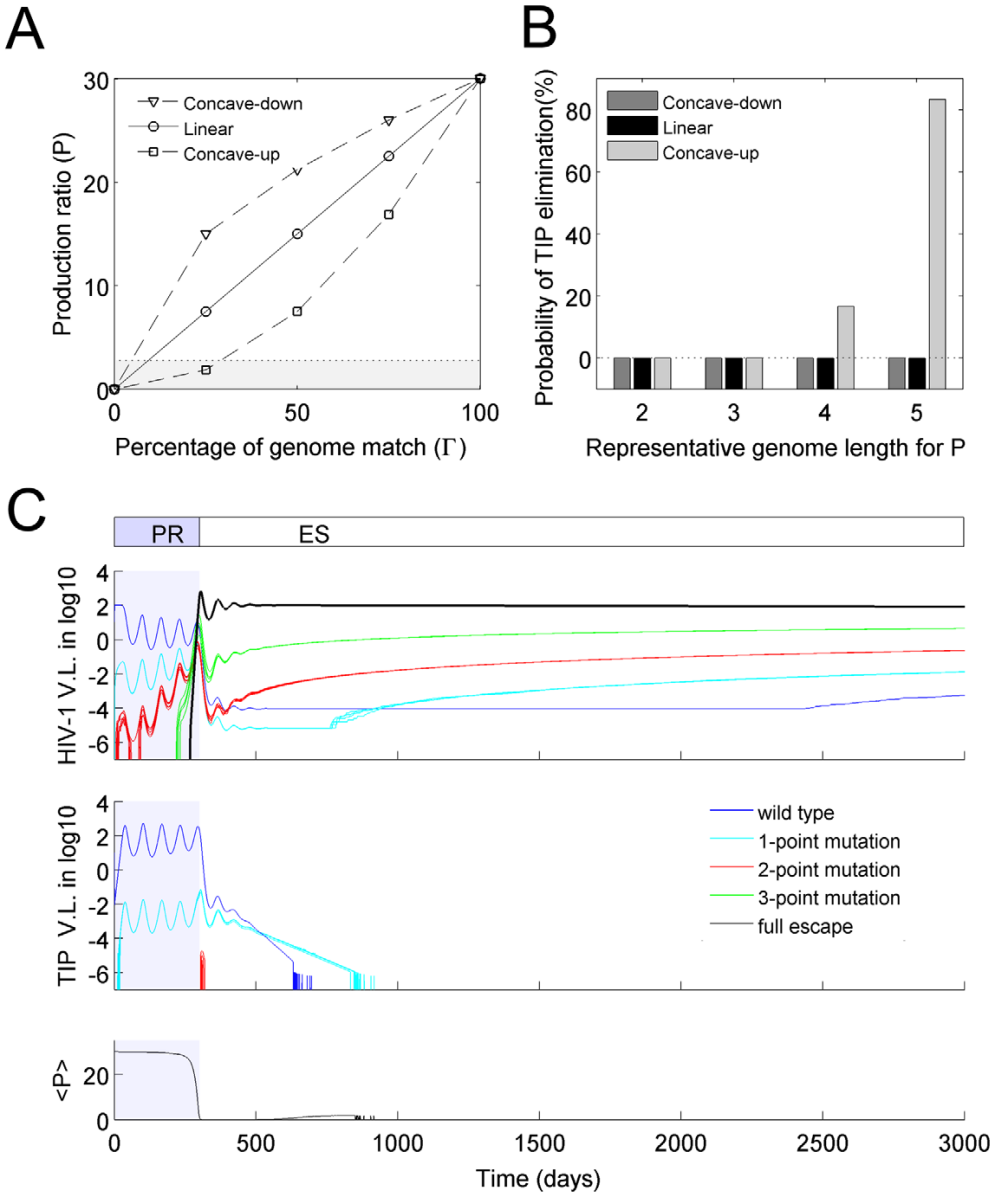
Effects of alternative genotype-phenotype mappings. (**A**) Different assumptions of genotype-phenotype mapping, showing how the production ratio parameter *P* depends on the proportion of sites that match on the relevant genome regions G_HP_ and G_TP_. The function form is 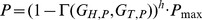. The values of the exponential constant are: *h = 0.5* for the concave-down curve in blue, *h = 1* for the straight line in red, *h = 2* for the concave-up curve in green. The black dotted line shows the minimum *P* value required for mutant TIP to invade the system (invasion threshold, *P*_threshold_). (**B**) The probability that TIP population was eliminated, under different assumptions of genotype-phenotype mapping (color-coding is same with (A)) for different lengths of the representative genome for parameter *P*. Each bar reflects the outcome of 100 runs of the multi-strain model. (**C**) A representative model realization showing the elimination of TIP. In this model, only variations in *P* are considered with parameters *A = 1* and *D = 0* fixed for all cells, and the length of representative genome is 4.

A fundamental challenge in modeling evolutionary processes is defining the genotype-phenotype relationship. For the current analysis, this is most important for determining the parameter *P* (phenotype) from the G_HP_ and G_TP_ regions of the HIV-1 and TIP genomes (genotype). In the results presented above, we assumed a linear relationship between the production ratio, *P*, and the percentage of match between the sequences of HIV-1 and TIP genomes (red line in Fig. 5A). Here we tested how the co-evolutionary dynamics are affected by two alternative assumptions about this genotype-phenotype mapping: the production ratio, *P*, is either a concave-down function or a concave-up function of the percentage of genomic match (blue and green curves in Fig. 5A).

In almost all scenarios, the co-evolutionary dynamics arising from these alternative genotype-phenotype maps were qualitatively similar to the model with a linear function (Fig. S2). In general, models assuming a concave-down function showed better TIP performance (longer time to HIV-1 escape, shorter duration of escape phase, and higher set-point <*P*>) than models assuming linear and concave-up functions (Fig. S2A). This occurred because the concave-down function gave higher *P* values for relatively poorly-matching genomes, favoring TIP in the co-evolutionary arms race. Accordingly, the average HIV-1 viral load was always lowest in models assuming a concave-down function. In contrast, for models assuming a concave-up function, it is possible for HIV-1 to escape control by TIPs completely, and for TIPs to be eliminated from the system, when the length of G_HP_ is 4 or greater (Fig. 5B).

A representative simulation showing the elimination of TIP is shown in Fig. 5C. The PR phase showed dynamics similar to those seen before (Fig. 3B), but after the full-escape HIV-1 mutant dominated the HIV-1 population at the beginning of the ES phase, the extant TIP strains (i.e. the wild-type and the one-point mutant strains) declined in abundance and eventually were eliminated from the system. This happened because the production rate of TIP in the dually infected cells was lower than the minimum required for persistence. We calculated the minimum threshold value of the production ratio to be *P_threshold_* = 2.80 (shown as dotted line in Fig. 5A). When the length of G_HP_ is 4 or 5 sites, both the wild-type TIP and the 1-point TIP mutant give *P*<*P_threshold_* when co-infecting a cell with the full-escape HIV-1 mutant (Fig. 5A), causing these TIP strains to decline to extinction. Note that low levels of 2-point TIP mutants are sometimes present at the beginning of the ES phase. In the simulation shown in Fig. 5C, the frequency of T cells infected by these 2-point TIP mutants was not high enough to be further infected by HIV-1 to become dually infected cells, as needed to complete the life-cycle of TIP. However, in some simulations the abundance of these 2-point TIP mutants was slightly higher due to the stochasticity of the system, which enables these mutants to complete their life cycle so that TIP persists in the host. As a generality, the ability of TIP to persist in the system depends on whether the TIP strains present at the onset of the ES phase are able to persist in a system dominated by the full-escape HIV-1 mutant. Under the assumptions of a concave-up function with representative genome length of more than 3, TIP extinction is possible because low *P* values arising from coinfection of cells with extant TIP strains and the full-escape HIV-1 strain.

In the simulations above, we assumed that HIV-1 and TIP mutate at the same rate and that mutations of HIV-1 and TIP change the phenotypic parameters in dually infected cells in the same way. The assumption of equal mutation rate is appropriate, since TIP genomes are replicated by the exact same mechanisms as HIV-1 genomes. However, since TIP interacts with HIV-1 in dually infected cells in a complicated way involving processes such as genome-protein binding, mutations in the TIP and HIV-1 genomes may impact differently on changes of the phenotypic parameters in dually infected cells. Focusing on the crucial interactions that determine the parameter *P*, one way that HIV-1 can escape TIP repression is by a mutation in the Tat gene which changes the Tat protein conformation. This change may lead to a lowered binding affinity for extant TIP genomes, thus giving the mutated HIV-1 strain a selective advantage over other strains. In order to regain the higher binding affinity, TIP needs to mutate the Tat-binding region on its genome. Hence, in this particular scenario, the parameter *P* is determined by mutations in HIV-1 and TIP that act at the amino acid and nucleotide levels, respectively. Therefore, the rate of change in parameter *P* could be differentially affected by mutations in the HIV-1 and TIP genomes. To account for this complexity, we examined the probability of TIP elimination as a function of the rate at which TIP mutation changes the crucial phenotypic parameter *P* relative to HIV-1 mutation. We found that the system is broadly robust to variation in the relative rate of phenotypic changes in HIV-1 and TIP (Fig. S3). When a concave-up function was used for the genotype-phenotype mapping, so that elimination of TIP is a possibility, the relative rate of evolution has a strong influence on the outcome in an intuitive manner: the probability of TIP elimination is lower when TIP mutates faster than HIV-1 in the phenotypic space, and vice versa (Fig. S3). When other genotype-phenotype mappings were used, the probability of TIP elimination was unaffected by relative mutation rate.

### Fitness costs of HIV-1 mutations

**Figure 6:**
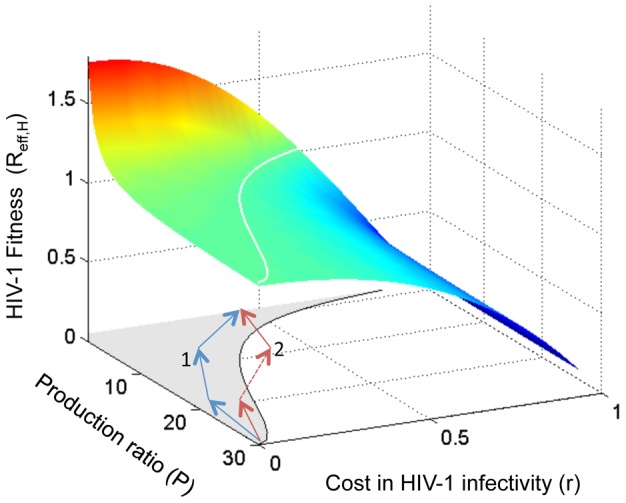
The fitness trade-off for HIV-1 if decreasing the production ratio, *P*, leads to reduction in viral infectivity. The surface shows the value of the effective reproductive number for an HIV-1 mutant with the given parameters invading a system with wild-type HIV-1 and TIP at equilibrium. The white line shows the combinations of parameters that give R_eff,H_ = 1. The shaded area shows parameter regions where the mutant HIV-1 has a higher fitness than the wild-type HIV-1 (R_eff,H_>1). Routes 1 and 2 (blue and red, respectively) show two different mutational trajectories by which the ‘full-escape’ mutant could be generated; route 2 is much less likely to be completed because it includes a fitness valley with lower fitness than the wild-type strain.

In the simulations above, we have assumed that HIV-1 mutants in the G_H,P_ region are as competent as the wild-type strain in terms of replication and infection of new cells. However, mutations in the HIV-1 genome change the properties of HIV-1 viral particles, and in general, they are likely to reduce viral replication and infectivity [61], [62], [63], [64]. If the fitness cost of these reductions is greater than the gain from lowering the production ratio, then HIV-1 mutations lowering the value of *P* would not be selected, i.e. HIV-1 would not be able to escape from TIP. We analyzed the impact of a mutation-induced reduction in HIV-1 fitness on the selection of the full-escape mutant. To make the analysis clear, we considered the scenario when HIV-1 mutants with mutations in the G_H,P_ region have decreased viral infectivity. Reduction in HIV-1 fitness arising from other mechanisms would lead to similar results. We performed invasion analysis for the mutant, and found a tradeoff between lowering *P* values and decreasing infectivity (Fig. 6). For a given decrease in *P*, if the corresponding reduction in infectivity is smaller than a threshold value (white line in Fig. 6), then R_eff,H_>1 and the mutant with lower *P* value is selected (the shaded area in Fig. 6); otherwise, the wild-type HIV-1 strain is maintained in the system. To confirm that mutant HIV-1 strains are not selected if the fitness reduction (via reduced infectivity) is too high, we simulated the multi-strain model under the simple assumption that all HIV-1 mutants have a 30% reduction in infectivity, with all other parameter values the same as in Fig. 5C. In stark contrast with the dynamics shown in Figs. 3C and 5C, the viral populations were dominated by the wild-type HIV-1 and the wild-type TIP throughout the simulation, and no escape events occurred (Fig. S4). The average *P* value remained fixed at 30, and the set-point HIV-1 viral load was reduced to 5 *virions/*µ*L*.

The surface shown in Fig. 6 can be viewed as a fitness landscape for HIV-1 mutants during the PR phase, given a tradeoff between decreasing *P* and maintaining HIV-1 infectivity. Generation of a full-escape mutant requires several mutational steps. If each step results in a fitness gain, i.e. moving uphill on the surface, then the full-escape strain can be generated by Darwinian selection (route 1 in Fig. 6). However, if one or more mutants along the mutational trajectory have lower fitness than their parents, i.e. deleterious mutations, then the generation of the full-escape mutant requires a low-probability event such as double mutation or stochastic tunneling to cross the fitness valley (route 2 in Fig. 6) [65], [66]. This suggests a design principle for TIPs: it is desirable to design TIPs so that HIV-1 must mutate its conserved genome region (which may induce a high fitness cost to HIV-1) to reduce the production ratio (*P*). In this way, the selection of HIV-1 escape mutants can be prevented due to the high cost associated with HIV-1 mutation. Interestingly, with regard to their effect on HIV-1 infectivity, the mutations along route 1 act synergistically to reduce the fitness of HIV-1 (i.e. exhibiting negative epistasis), while the mutations along route 2 act antagonistically (i.e. exhibiting positive epistasis). Previous work has shown that the majority of deleterious mutations act antagonistically in HIV-1, i.e. with positive epistasis [67]. This property of HIV-1 genetics suggests that the generation of a full-escape mutant of HIV-1 may be constrained by the current design of TIPs.

## Discussion

In this study, we have used mathematical models to analyze the co-evolutionary dynamics of HIV-1 and a gene therapy delivered by a conditionally-replicating vector (CRV) in the peripheral blood within a host. We have considered the proposed therapeutic interfering particle (TIP) as a case study for this analysis, which has enabled us to build on recent modeling studies, and has provided specificity and context for our findings. We have investigated questions about HIV-1 escape mutants and virulence evolution, and the potential to achieve long-term viral suppression despite viral evolution, as raised in recent CRV studies [19], [21], [30], [35].

### Co-evolution of HIV-1 and TIP

Linking models describing dynamics at both the cellular and the within-host level, we have shown that, under most conditions, the TIP strategy is able to circumvent the evolution of resistance by HIV-1. The TIP population is able to keep pace with the evolution of HIV-1, and thus maintains effective suppression of the HIV-1 viral load in the long-term. The long-term dynamics of HIV-1 and TIP have characteristics of a co-evolutionary arms race (also termed as ‘Red-Queen’ dynamics) [68]. HIV-1 mutants that escape TIP suppression have a fitness advantage and rise in frequency, leading to selection for TIP mutants that ‘catch up’ and can suppress the HIV-1 mutants. For the broad range of parameter values and model structures that we analyzed, this cycle of escape and catch-up continues indefinitely, and the TIP population results in long-term control of the HIV-1 infection within a host. This finding points to the potential for a new generation of CRV-delivered gene therapy agents, which co-opt the viral evolutionary process to design robust and ‘evolution-proof’ disease control.

This distinctive pattern of co-evolutionary dynamics is driven by selection on the production ratio, *P*, which describes the fold increase in genomic RNA production for TIP relative to HIV-1. The invasion analysis shows that selection on this parameter acts in opposing directions for the HIV-1 and TIP populations in a co-infected host (Fig. 2). TIP benefits from high values of *P*, while HIV-1 benefits from low values and hence is under selection to acquire substitutions that escape TIP by decreasing the degree of genome matching. Then the TIP population is under selection to catch up with HIV-1 mutation to restore a high *P* value. This arms race underlies the characteristic three-phase dynamics that arise as a robust pattern in our co-evolutionary simulations (Fig. 3). In the preliminary phase at the onset of treatment, HIV-1 mutants appear and rise in frequency due to the lower *P* they experience. Soon a ‘full-escape’ HIV-1 mutant appears, which experiences no suppression by the wild-type TIP, and rises to dominate the population throughout the escape phase. For most scenarios we considered, this is followed by the set-point phase where the TIP population is able to mutate to match the HIV-1 strains, and sustained suppression of the HIV-1 viral load is achieved.

We explored the circumstances under which TIP could not establish a set-point phase, and found that the TIP population could be eliminated when two conditions are met. First, any fitness cost associated with HIV-1 mutations must be sufficiently low that the HIV-1 mutational steps to escape the repression of TIP are always increasing in fitness (Fig. 6). Second, once the full-escape HIV-1 mutant takes over the population, the production ratios (*P*) in cells dually infected by extant TIP strains and the full-escape HIV-1 must be below the invasion threshold, i.e. the production of TIP particles is too slow to sustain the TIP population (Fig. 5C). In addition, we find that when elimination of TIP becomes possible, the probability of elimination is decreased if TIP mutations change the parameter *P* faster than HIV-1 mutations, and vice versa.

Our analysis reveals particular design principles that would enhance the efficacy and safety of TIPs, and we propose that similar principles would apply to other CRV gene therapies. Consideration of the factors that enable TIP to persist leads to the finding that the mapping between genotype and phenotype for the production ratio has paramount importance for the efficacy of TIPs. In simulations with a concave-down curve for the mapping (Fig. 5A), i.e. a lower reduction in *P* for intermediate mutants that lead to the full-escape mutant, a lower HIV-1 viral load is observed relative to simulations with either a linear or a concave-up curve (Fig. S2A). Therefore, a high priority in designing the TIP must be that it maintains a high *P* value when it coinfects T cells with HIV-1 strains that have acquired a few escape mutations (i.e. so the TIP and HIV-1 genome regions corresponding to *P* match partially). A related conclusion is that design principles that increase the minimum value of *P*, such that even full-escape mutants of HIV-1 do not reduce *P* below the critical value *P_threshold_*, will lead to much greater evolutionary robustness.

Another facet of the proposed TIP design is to encode an inhibitory factor that interferes with the HIV-1 life cycle to reduce HIV-1 viral loads [46], [47]. However, the model analysis shows that both HIV-1 and TIP attain maximal fitness when this factor is non-functional, i.e. with no inhibition (*D* = 0; Fig. 2). This leads to the prediction that both populations will evolve to diminish the activity of the inhibitory factor, which is borne out by simulation results in the multi-strain co-evolutionary model (Fig. 3B). Since HIV-1 viral load can be suppressed when the inhibitory factor is non-functional [30], we propose that the inhibitory factor is not needed for the TIP design. Note that earlier modeling work predicted that mutants that upregulate HIV-1 gRNA production in dually infected cells (i.e. those with *D*<0) are selectively more advantageous than mutants with no inhibition (*D* = 0) [30], which differs from our results here. This discrepancy arises because we added the assumption, based on experimental evidence [52], [53], that elevated HIV-1 gRNA production induces costs to HIV-1 fitness by reducing T cell lifetime.

The possible adverse consequences of co-evolution are considered by analyzing the selection exerted by the presence of TIP on the replication coefficient *A* of HIV-1. Our analysis suggests that the optimal genome replication rate of HIV-1 is dependent on how the infected cell lifetime changes as the HIV-1 genome replication increases, i.e. the function *Ω*. Under our model formulation, if the death rate of HIV-1 infected cells increases linearly with increases in HIV-1 replication, HIV-1 intra-host fitness remains the same and the presence of TIP does not select for HIV-1 strains with higher replication rate. If the death rate of HIV-1 infected cells increases non-linearly with increases in HIV-1 replication (as assumed in our main analyses), and the curvature *α* of this relationship is moderate or stronger, then the optimal HIV-1 replication rate stays near the wild-type level in the presence of TIP. However, if the curvature is weak, so that HIV-1 intra-host fitness decreases only slightly as HIV-1 replication rate increases beyond the wild-type level, then the optimal rate of HIV-1 replication can be higher than the wild-type rate in the presence of TIP. Empirical evidence for the relationship between HIV-1 replication rate and infected cell lifetime is not conclusive. Some indirect evidence suggests that higher viral replication rates incur a significant cost in cell lifetime [52], [53]; however, one study reported that HIV-1 replication induces little cytopathic effect on host cells [69], and two other studies showed that the death rate of HIV-1 infected cells appears to be unaffected by the presence of cytotoxic CD8^+^ T cells [70], [71]. Further experiments examining how HIV-1 fitness changes with variation in the HIV-1 genome replication rate would enable more precise predictions.

### Model assumptions and limitations

As for all models, we made simplifying assumptions during model construction. Most importantly, the representations of the HIV-1 and TIP genomes, and the relationship between these genotypes and the resulting cellular phenotypes, are highly simplified. In the multi-strain co-evolutionary model, each genome is represented by a digital sequence, and mutations in the genomes are mapped to changes in phenotype (i.e. the values of parameters *D*, *P* and *A*) via simple matching algorithms. We have ignored epistatic interactions and assumed that mutations affect the phenotypic parameters additively. In reality, changes in phenotype are affected by viral mutations in complicated ways, which often are not understood completely; this is necessarily the case for therapies like TIP that are still hypothetical. However we note that the digital sequences in our model are an abstract representation of any information encoded in the genome, and are not restricted to representing a particular set of nucleotide loci. Hence any genome properties that influence the phenotype of interest can be represented. To test the robustness of our conclusions, we performed extensive sensitivity analyses for different parameter values, genome structures, genotype-phenotype mappings and superinfection rates. The results show that the qualitative behavior of the system does not depend on these assumptions, beyond the broad findings discussed above. Other factors recently shown to influence the infection-limiting effects of defective interfering particles, such as dose-dependent responses [26], host cell limitation [72], and potential synergy with the host immune response [73], should be explored in future work.

The assumptions pertaining to the parameter *P* merit special attention, given that parameter's central role in the co-evolutionary dynamics. We have assumed that *P* is determined by the degree of matching between relevant regions on the HIV-1 and TIP genomes. This assumption is motivated by the necessary interactions between the TIP genome and HIV-1-encoded elements that regulate genome replication [37]. However, some factors proposed to contribute to TIP gRNA over-expression, such as TIP's shorter genome length or re-engineered splice sites [30], may not be influenced directly by the HIV-1 genotype present in the cell. If these factors do act to increase *P* in a manner independent of HIV-1, then they will have the effect of raising the minimum value of *P* corresponding to a full-escape mutant. As noted above, this will benefit TIP in the co-evolutionary process, making it more robust to elimination and HIV-1 escape. Our assumption that *P* is fully controlled by the interaction between genomes is thus conservative with respect to estimating the benefits of TIP.

In our model, TIP competes with HIV-1 for resources through dimerization with HIV-1 gRNA, and we have assumed that TIP and HIV-1 gRNAs dimerize randomly in dually infected cells. Under this assumption, the efficacy of TIP would be undermined if the DIS of HIV-1 or TIP mutates to reduce the rate of heterodimer formation. Our analysis showed that, if the cost of DIS mutation to viral fitness is greater than the gain from lowering the rate of heterodimer formation, then mutations in the DIS are unlikely to arise. Currently available experimental data support this conclusion (see Text S1). However, the margin of safety in these results was small, so if fitness costs for TIP are less than projected or if other mechanisms favor mutations in the DIS region, then this could be a vulnerability of the proposed TIP strategy. We suggest that robustness of heterodimer formation be a focus of on-going research on TIP design. Further experimental investigation is needed to test the stability of the DIS regions of the HIV-1 and TIP genomes in dually infected cells, and alternative approaches to increasing TIP production relative to HIV-1 should be explored.

In the model, we have only considered infection of productively infected T cells in the peripheral blood. There are two other sites of HIV-1 production that can play important roles in the dynamics of infection: long-lived cells and tissue cells. It has been shown that long-lived cells may have a significant influence on viral dynamics during the chronic phase of infection [74]. Modeling both short- and long-lived cells is beyond the scope of our study, but we have performed simulations for models considering only long-lived cells, and found that the three-phase dynamics are robust to this change though they occurred on a longer time scale (data not shown). Infections of tissue cells pose greater challenges. Data show higher multiplicity of infection for HIV-1 in tissue cells, probably resulting from formation of virological synapses [75]. Our model does not consider cells infected with multiple strains of HIV-1 or of TIP, since the intracellular interactions among different strains of HIV-1 and TIP are not currently understood. Intuitively, the majority of infecting viruses in these multiply-infected cells would be TIPs, because of the much higher viral load of TIP. We speculate that this would lead to broader and stronger selective pressure on HIV-1 due to the presence of different TIP variants within the cell. Therefore, the period of the ‘preliminary’ phase would be shorter, and the three-phase dynamics would remain. However, we emphasize that there are fundamental uncertainties about how these processes would play out, and the genetic exchange between viral populations in tissues and in peripheral blood will make the co-evolutionary dynamics more complex. Because a substantial amount of HIV-1 replication occurs in tissues, these dynamics could have significantly impact on the prediction of sustained control of infection in a patient. This prediction should be evaluated further using models that explicitly consider multiply-infected cells in tissue, and the interactions between viral populations in different body compartments, once the biological processes are understood in sufficient detail.

### Broader implications

The evolutionary principles developed in this study, including the robust pattern of three-phase dynamics of evolutionary escape and catch-up, provide general lessons for other CRV-based gene therapies against HIV-1. All CRVs transmit from cell to cell via the same basic mechanism of complementation with viable viral genomes, and therefore the within-host model is a general representation of the population dynamics of CRV and HIV-1 strains in a patient. The cellular model was constructed based on the proposed properties of TIPs in particular, but is easily related to other systems by noting its key outputs. As shown in the Methods, the two quantities that link the cellular level to the within-host level are the HIV-1 production rate in both singly and dually infected cells, and the CRV production rate in dually infected cells. These are the fundamental properties of any CRV or mobilization-competent gene therapy system.

From a conceptual perspective, the various strategies of constructing CRVs differ only in how these key quantities change depending on the molecular mechanisms of CRV replication and interference with HIV-1. The evolutionary principles regarding the competition and inhibition between CRVs and the HIV-1 population at the within-host level are the same. Our findings, in conjunction with those of Weinberger *et al.* [37] and Metzger *et al.* [30], show that any CRV-based approach has potential to achieve sustained control of an HIV-1 infection if it has the following traits: 1) the CRV is replicated at a sufficiently high level in dually infected cells that it can be persistently transmitted within a host; 2) the CRV competes with HIV-1 for essential resources required for replication and packaging, creating an evolutionary conflict that leads to a co-evolutionary arms race between HIV-1 and CRV; 3) the CRV is designed such that a) it targets a conserved region of HIV-1, and consequently HIV-1 escape mutants cannot be generated due to the high fitness cost, or b) when the full-escape mutant takes over the HIV-1 population, the extant CRV strains are able to catch-up with the full-escape HIV-1, by replicating at a sufficiently high level in cells infected by the full-escape mutant. These general conclusions suggest design principles to ensure the evolutionary robustness of viral gene therapies based on CRVs.

In addition, due to the similarities between CRVs and naturally-occurring defective interfering particles, the results in this study also shed light on the dynamics of defective interfering particles observed both in experimental studies [31] and in natural populations [76]. Earlier theoretical studies have examined the effect of defective interfering particles on viable viral populations [72], [77], [78], [79]. In particular, Kirkwood and Bangham developed a mathematical model to understand the evolutionary dynamics of a wild-type virus with its associated defective particles in serial passage experiments, and concluded that the effects of defective particles were intrinsically unpredictable [77]. However, their model assumed that the defective interfering particles were generated constantly from the extant viable viral population and only interfered with their parent strains. Mutants generated within lineages of defective interfering particles, and the potential impacts of interference between multiple strains, were not considered in their model. By accounting for the possibility of continuous evolution in the interfering particle population, our study shows that the system can approach a sustained co-evolutionary arms race as observed experimentally [32].

The rapid evolution of HIV-1 poses fundamental challenges for all strategies of treatment and prevention. In the long term, HIV-1 evolution can compromise the efficacy of these treatments and even render them useless. This phenomenon is well-known for ARV drug therapies [6], [44] and vaccine candidates [8], but is increasingly recognized for gene therapies as well. For example, Aviron *et al.* recently analyzed the evolutionary dynamics of HIV-1 for another class of gene therapy approaches, in which T cells are genetically modified such that they confer resistance to HIV-1 infection and replication [50]. The dynamics of HIV-1 under this gene therapy show many parallels with the dynamics of HIV-1 under other non-evolving therapies, such as ARVs. During the initial period of the treatment, the wild-type HIV-1 remains the dominant strain, and the viral load is effectively suppressed. More and more HIV-1 mutants are generated, until the full-escape mutant appears and quickly takes over the virus population. Once the full-escape mutant arises, the genetically modified T cell does not exert its protective effects anymore, and HIV-1 returns to its pre-treatment abundance [50]. Our study suggests that gene therapies based on CRVs, such as the proposed TIP, could continue to inhibit HIV-1 viral production by keeping pace with HIV-1 evolution even after the full-escape mutant is generated. This phenomenon, which mirrors the co-evolutionary dynamics of an immune system, stands in stark contrast with drug treatments and ‘static’ gene therapy approaches [44], [50]. In addition, in the modeling study by Metzger *et al.*, it has been shown that CRVs can potentially act synergistically with ARVs [30]. Since ARVs and CRVs target different components of the HIV-1 life cycle, it is likely that a combination of ARV and CRV therapies would reduce the risk of generation of HIV-1 mutants that escape ARVs and CRVs. Furthermore, our results suggest potential for rational design of gene therapies based on conditionally-replicating vectors to avoid undesirable evolutionary outcomes—in keeping with recent calls to construct ‘evolution-proof’ approaches to disease control [80]. By harnessing the remarkable evolutionary potential of CRVs in this way, this new class of gene therapy agents could contribute a valuable new dimension to the increasingly successful effort to combat the HIV-1 pandemic worldwide.

## Methods

### The intracellular model

Following the framework proposed by Metzger *et al.* [30], the intracellular model keeps track of the abundance of single-stranded HIV-1 and TIP genomic RNAs, as well as dimerized diploid genomes including the HIV-1 homozygote, the TIP homozygote, and the HIV-1-TIP heterozygote. We assume that the level of single-stranded HIV-1 gRNA reaches equilibrium quickly, and that packaging materials are present in excess [30], so the dimerization of genomic RNAs is the limiting step in the process of viral particle formation. Thus, the rate of formation of new HIV-1 virions can be approximated as proportional to the rate of RNA dimerization. The detailed model equations are presented in the Text S1.

We define two parameters, *ψ* and *ρ* to link the intracellular model to the within-host model, as in previous work [30]:

1. *ψ* is the ratio of the HIV-1 viral production rate in a dually infected cell over the rate in an HIV-1 infected cell.
2. *ρ* is the ratio of the TIP viral production rate over the HIV-1 viral production rate in a dually infected cell.

The expressions for *ψ* and *ρ* are related to intracellular parameters by [30]:
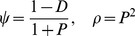
(1)where *P* is the production ratio for genomes and *D* is the fraction of inhibition, as defined in the main text. The replication coefficient *A* is the ratio of the HIV-1 replication rate for a mutant genotype to the replication rate of the wild-type HIV-1 genotype. According to Eqn. (1), the value of *ψ* decreases with increases in *D* and *P*, because either stronger inhibition on HIV-1 gRNA production or higher CRV production that competes with HIV-1 production will lead to lower HIV-1 viral production. The value of *ρ* depends on the value of *P*, but not *D*. This is because inhibition of HIV-1 gRNA production limits the gene products available to both HIV-1 and CRV, therefore changes in *D* affect HIV-1 and CRV production similarly. For an arbitrary HIV-1 genotype, the production rate of HIV-1 virions in dually infected cells, relative to the production rate of wild-type HIV-1 in singly-infected cells, is *Aψ*. The production rate of TIP virions in dually infected cells, relative to the production rate of the wild-type HIV-1 in singly-infected cells, is then *Aψρ*.

Note that the parameters *P*, *A*, *D*, *ψ* and *ρ* are subscripted in the multi-strain model below in order to specify the value for particular HIV-1 and/or TIP strains. For example, *A_0_* denotes the *A* value for the wild-type HIV-1 (‘0’ is used throughout to denote the wild-type), and *P_01_* denotes the *P* value in a cell dually infected by the wild-type HIV-1 and the 1^st^ TIP mutant strain.

### The general within-host model

To model the dynamics of viral populations within individual hosts, we consider the infection dynamics of CD4^+^ T cells by *m* strains of HIV-1 and *n* strains of TIP. We assume that each T cell can only be infected by a single strain of HIV-1 and a single strain of TIP. The dynamics are described by the following system of ODEs:
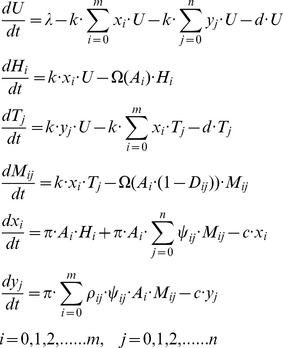
(2)Uninfected T cells (*U*) are generated at a constant rate *λ*, and cleared from the blood at rate *d*. The *i^th^* HIV-1 and *j^th^* TIP strains infect T cells at rate *k*, resulting in HIV-1 infected T cells (*H_i_*) and TIP infected T cells (*T_j_*), respectively. TIP infected T cells can be further infected by HIV-1, becoming dually infected cells (*M_ij_*). Because TIP genomes do not encode any protein that is toxic to the cell or that induces immune response, the death rate of TIP infected cells is assumed to be the same as the death rate of uninfected cells. The death rates of HIV-1 infected cells and dually infected cells are modeled as a concave-up function (*Ω*(), described below) depending on the HIV-1 genomic RNA (gRNA) production rates (*A* in singly infected cells and *A*(*1-D*) in dually infected cells as shown in Eqn S1 and S3 in Text S1).

Viral particles of the *i^th^* HIV-1 strain (*x_i_*) are produced from both singly and dually infected cells, whereas viral particles of the *j^th^* TIP strain (*y_j_*) are produced only from dually infected cells. The rate of HIV-1 viral production in cells infected only with wild-type HIV-1 is *π*. The rates of viral production in cells infected with mutant HIV-1 strains, and in dually infected cells, are scaled relative to *π* by the relationships given in the previous section on intracellular dynamics. Both HIV-1 and TIP particles are cleared from the system at a constant rate, *c*.

#### Dependence of cell death rate on virion production

The accumulation of HIV-1 viral proteins within a cell and the depletion of host resources due to HIV-1 replication have cyto-toxic effects leading to increased death rate of infected T cells [52], [53]. In addition, the number of viral peptides presented at the cell surface increases as viral protein production rises, triggering the immune response of the host [52]. All of these factors likely contribute to an accelerating increase of cell death rates as HIV-1 viral protein production increases. We therefore assume that the death rate of HIV-1 infected and dually infected T cells is a concave-up function *Ω*(*A*) of the production rate of HIV-1 genomic RNA. This assumption follows models in previous studies [54], [55]. In the absence of TIP, this formulation leads to an optimal HIV-1 replication rate, i.e. the wild-type production rate *π* [81].

Specifically, we modeled the function *Ω()* using the following equation:
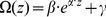
(3)where *z* is the production rate of HIV-1 gRNAs in infected cells (relative to wild-type HIV-1 in singly-infected cells), *α* reflects the stiffness of the curve and *β* and *γ* are constants that reflect biological properties of the death of infected cells. In most of our analyses, we set *α = 1*, but we conduct a sensitivity analysis where the value of *α* is varied (Fig. S1). The function *Ω()* must satisfy two biological conditions: 1) the death rate of T cells infected by the wild-type HIV-1 is 0.7 per day [12], [13]; and 2) the wild-type HIV-1 has optimal replicative fitness in the absence of TIP. These two conditions translate into the following mathematical statements:

1. *Ω(1) = 0.7*. Note that *A* = 1 for wild-type HIV-1 infected cells.
2. *A/Ω(A)* attains its maximum value at *A* = 1. The expression, *A/Ω(A)*, is proportional to the burst size of HIV-1 infected cells, which is optimized for wild-type HIV-1 in the absence of TIP [81].

The values of *β* and *γ* can be calculated under these two conditions for a given value of *α*, as shown in Table 2.

**Table 2:** Description of parameters used in the model.

Parameter	Description	Value	Source
**λ**	Birth rate of uninfected CD4+ T cells (U)	31 [cells/(µL×day)]	[82]
**D**	Death rate of uninfected CD4+ T cells (U)	0.02 [1/day]	[83]
**k**	Infection rate of activated CD4+ T cells per virion	1.875×10^−4^ [µL/(virions×day)]	[30]
**Λ**	Production rate of virions released from HIV-1 infected cells	140 [1/day]	[84]
**C**	Clearance rate of HIV-1 (x) and TIP (y) virions	30 [1/day]	[85], [86]
**μ**	Genome mutation rate	3×10^−5^ per nucleotide	
**α**	Stiffness of cell death rate curve	1†	
**β**	Constant in cell death rate function	0.2575††	
**γ**	Constant in cell death rate function	0††	
**A**	*Replication coefficient*. The relative rate of gRNA replication of a HIV-1 strain compared to the wild-type in the absence of TIP.	0.5–1.5	
**P**	*Production Ratio*. The ratio of TIP gRNA production over HIV-1 gRNA production in a dually infected cell (M).	0–30	†††
**D**	*Fraction of inhibition* (D>0) or upregulation (D<0). The inhibition/upregulation of HIV-1 production exerted by the inhibitory factor encoded by TIP. When D = 0, the factor is non-functional	−1–1	†††

† Sensitivity analyses were performed on this parameter (Fig. S1). Choice of this parameter value does not affect qualitative results in this study.

†† See Methods section for the derivation of parameter values.

††† Parameters that can be genetically designed.

#### The minimum value of *P* for TIP persistence, *P_threshold_*

The value of *P_threshold_* is determined as the smallest value of *P* that allows TIP to persist in a HIV-1 infected host. We first derive the reproduction number for TIP (R_0,T_) when it is introduced in a host where HIV-1 is at equilibrium. It is calculated using the next-generation matrix approach for a system in which only HIV-1 is present at equilibrium. The expression for R_0,T_ is:
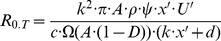
(4)where x′ and U′ are the equilibrium level of HIV-1 virions and uninfected T cells, respectively, in the absence of TIP.

Since the requirement for TIP to persist in the system is R_0,T_>1, the value of *P_threshold_* can be calculated by solving the value of *P* for R_0,T_ = 1 given Eqn. (4). If we assume *D = 0*, *A = 1*, i.e. the optimal values of D and A (Table 1), the value of *P_threshold_* is 2.80.

#### The HIV-1 mutant model

The HIV-1 mutant model is a simplified version of the General Model, which considers only the wild-type HIV-1, the wild-type TIP and a mutant HIV-1 strain. The full ODEs are listed in Text S1 (Eqns. S7). To analyze whether the HIV-1 mutant strain is able to invade a system with the wild-type HIV-1 and wild-type TIP at equilibrium, the effective reproductive number for the HIV-1 mutant (R_eff,H_) can be calculated using the next-generation matrix approach [51]:
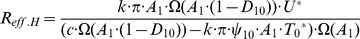
(5)where U* and T_0_* are, respectively, the equilibrium levels of uninfected T cells and T cells infected by the wild-type TIP in the absence of the HIV-1 mutant. This expression has been confirmed by simulation to exhibit the expected threshold behavior where the HIV-1 mutant strain can invade only if R_eff,H_>1.

#### The TIP mutant model

Similarly, the TIP mutant model is a simplified version of the General Model, which only considers wild-type HIV-1, wild-type TIP and a mutant TIP strain. The full ODEs are listed in Text S1 (Eqns. S8). The effective reproductive number for the TIP mutant (R_eff,T_) is derived assuming the wild-type HIV-1 and wild-type TIP are at equilibrium before introducing the TIP mutant:
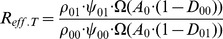
(6)Once again, this expression has been confirmed by simulation.

#### The modified HIV-1 mutant model – adding costs in infectivity

In the modified HIV-1 mutant model, we assume that there is a cost (*r*) in viral infectivity for the mutant HIV-1 strain. The reduced viral infectivity is modeled as (1-r)*k, where *k* is the wild-type infectivity. The other components of the model follow the HIV-1 mutant model described above, and in Eqns. S7. For this analysis, we considered changes in parameters *P* and *r*, and set *A = 1* and *D = 1*. The effective reproductive number for the HIV-1 mutant in this modified model (R_eff,mH_) is:
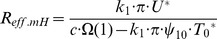
(7)where, as before, U* and T_0_* are the equilibrium levels of uninfected T cells and the T cells infected by the wild-type TIP in the absence of the HIV-1 mutant, respectively.

#### The multi-strain model

In the multi-strain model, each HIV-1 or TIP strain is represented by a unique genome sequence. The genomes of HIV-1 and TIP determine the intracellular parameters *A*, *P* and *D*. Mutations in the genome sequences result in changes in intracellular parameters, which in turn lead to changes in the *ψ* and *ρ* parameters linking the intracellular model and the within-host model. The dynamics of the multiple strains of HIV-1 and TIP within hosts are modeled by a stochastic-deterministic hybrid approach, combining ordinary differential equations (ODEs) with stochastic events [41].

The genome sequences of HIV-1 and TIP are represented as having *u* and *v* variable sites, respectively. For our main analysis the dimension of the sequences is set to 2, i.e. each site in a sequence can take two values, 0 or 1. To assess the sensitivity of the results to this assumption, the genome dimension is set to 3 or 4 in the sensitivity analysis shown in Fig. 4. The intracellular parameters *P*, *D* and *A* are calculated from the genomic sequence as follows.

The parameters *P* and *D* arise from interactions between the HIV-1 and TIP genomes or their gene products, so we calculated their values using a very simple model that assesses the degree of matching between the two genomes. We first define a function, *Γ(S_1_,S_2_)*, to be the proportion of sites in genome sequences *S_1_* and *S_2_* that are identical. Then, the parameter *P* in a dually infected cell is calculated as:
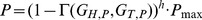
(8)where *G_H,P_* and *G_T,P_* are the genome regions that determine parameter *P* in the HIV-1 and TIP genomes, respectively, and *h* is the exponent determining the shape of this genotype-phenotype mapping. For Figs. 3 and 4 *h* is set to 1; for Fig. 5, it is set to 0.5, 1 and 2.

Similarly, the parameter *D* in a dually infected cell is calculated as:
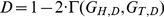
(9)where the factor *2* allows *D* to be negative, such that the TIP up-regulates the production of HIV-1 genomic RNAs, and the dependence on *Γ* is assumed to be linear. In this model, *D* can vary in the range of *[−1,1]*.

The parameter *A* is determined by the HIV-1 genome only, and is defined relative to the replication rate of the wild-type HIV-1 strain. We calculated this parameter as:
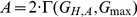
(10)where the factor *2* allows *A* to be greater than 1, i.e. the mutant can have a higher replication rate than wild-type HIV-1 (though recall from the section on cell death rates that this does not necessarily mean higher fitness). *G_Max_* is the sequence that gives the highest HIV-1 gRNA replication rate (*A = 2* in this study). We set the *G_Max_* is [1,1,1,1] and the *G_H,A_* for the wild-type HIV-1 is [0,1,0,1].

In the model simulation, we assume the levels of uninfected T cells, wild-type HIV-1 infected T cells and wild-type HIV-1 virions are in equilibrium before introducing TIP. The wild-type TIP is introduced in the ODEs on day 0. For each time interval (*Δt* = 1 day), the ODE system is numerically integrated, and then mutant strains are generated as stochastic events. We assume that for each event where a T cell is infected by HIV-1 or TIP, every position of the HIV-1/TIP genome has a probability *μ* to mutate. Since the probability of generating a double mutation for a short sequence space (*u* and *v<20*) in one day is extremely low, only single mutations are considered in the model. We first approximate the numbers of T cells newly infected by the *i^th^* HIV-1 strain (*ΔH_i_*) and the *j^th^* TIP strain (*ΔT_j_*) during the time interval, *Δt*, by
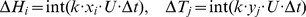
where the function *int(m)* returns the biggest integer number that is less than *m*.

We then calculated the number of newly infected cells in which the *i^th^* HIV-1 strain mutated to the *a^th^* HIV-1 strain (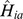) by drawing a random number from the binomial distribution, 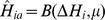. Similarly, we calculated the number of cells newly infected with TIP in which the *j^th^* TIP strain mutated to the *b^th^* TIP strain (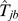) by drawing a random number from the binomial distribution, 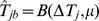. The copy numbers of the existing infected T cells are then updated as:
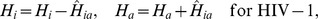


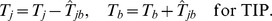
If the newly-generated mutant strain does not exist in the ODEs, it is added into the ODE system with the appropriate initial condition.

Extinction of rare strains was modeled using a quasi-extinction approximation. To implement this, we checked the abundance of each variant of infected T cell on each day. When the copy number of a particular variant is below 10^−6^ *copies/*µ*L* (roughly 1 to 10 copies of virions in a host), this variant was removed from the ODE system. For each simulation, this procedure was repeated for a total of 3000 days (approximately 8 years).

## Supporting Information

Figure S1
**The optimal values of parameters** ***A*** **and** ***D*** **do not vary significantly with variation in the curvature of the cell death function,** ***Ω()*** **, or production ratio** ***P*** **.** (**A**) Different shapes of the function ***Ω***(*A*), which describes the dependence of T cell death rate on the production rate of HIV-1 gRNA (*A*), used for sensitivity analysis. The solid black line denotes a linear function. Other lines arise from the function 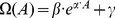 with *α* values shown in the legend and the values of *β, γ* were chosen such that ***Ω***(*1*) = 0.7. (**B**) The ranges of variation in optimal replication coefficient, *A*, and fraction of inhibition, *D*, under different assumptions of the cell death function ***Ω***(*A*) shown in panel (A). Each line is color coded according to the assumptions on ***Ω***(*A*) shown in panel (A). The optimal combinations of *A* and *D* are calculated with *P* varying from 5 to 30 (lines in the figure; circles correspond to values *P* = 5, 10, 20, 30, from left to right). The dashed lines show the range of variation in optimal *A* and *D* for each assumption on ***Ω*** *(A)*; the red shaded area shows the range of variation for the functional form (*α* = 1) used in other simulations.(TIF)

Figure S2 **Co-evolutionary dynamics are qualitatively similar for models with alternative assumptions of genotype-phenotype mapping, although models assuming a concave-down function show better TIP performance.** Five attributes are used to characterize the co-evolutionary dynamics of HIV-1 and TIP: the average HIV-1 and TIP viral loads (panels (A) and (B)) over the whole simulation (3000 days), the lengths of the PR phase and the ES phase (panels (C) and (D)) and the time-averaged mean value of *P* (panel (E)) during set-point phase. The values of *D* and *A* in these simulations are set to 0 and 1, respectively. 100 runs are performed for each data points. Data points and error bars correspond to the mean and standard deviation of 100 realizations of the model for those parameter values. Note that large standard deviations in data points for the concave-up function are due to TIP elimination events.
(TIF)

Figure S3 **Effect of variations in the relative rate that TIP and HIV-1 mutations change the phenotypic parameter** ***P*** **.** The probability of TIP elimination decreases as the relative rate of TIP evolution increases, when a concave-up genotype-phenotype mapping function is used. When the linear or concave-down mapping functions are used, the relative rate has no effect on TIP elimination. We represented the different rate at which mutations impacted the phenotypic parameter *P* by varying the mutation rate of the TIP genome in our genome-matching model. The ratios of TIP mutation rate over the HIV-1 mutation rate considered are 1/5, 1/2, 1, 2 and 5 in the simulations. For each ratio, 100 model runs were performed and the proportion of runs that exhibited TIP elimination is shown. Parameters *A* and *D* were kept constant at 1 and 0, respectively, in these simulations.
(TIFF)

Figure S4 **HIV-1 mutants are not selected when the cost of HIV-1 mutation is high.** In this simulation, we assumed that the infectivity of all HIV-1 mutants is reduced by 30% compared to the wild-type infectivity. This assumption leads to reduced fitness for those HIV-1 mutants that are intermediate steps to the full-escape mutant as shown in Fig. 6. All other parameters are the same as in Fig. 5C.
(TIFF)

Text S1 **Supplementary material.**
(PDF)
