# Supplementary figures and images for: Evolutionary Analysis of Human Immunodeficiency Virus Type 1 Therapies Based on Conditionally Replicating Vectors
Source: PLoS Comput Biol. 2012 Oct 25;8(10):e1002744. doi: 10.1371/journal.pcbi.1002744 (PMC3486895; doi:10.1371/journal.pcbi.1002744)

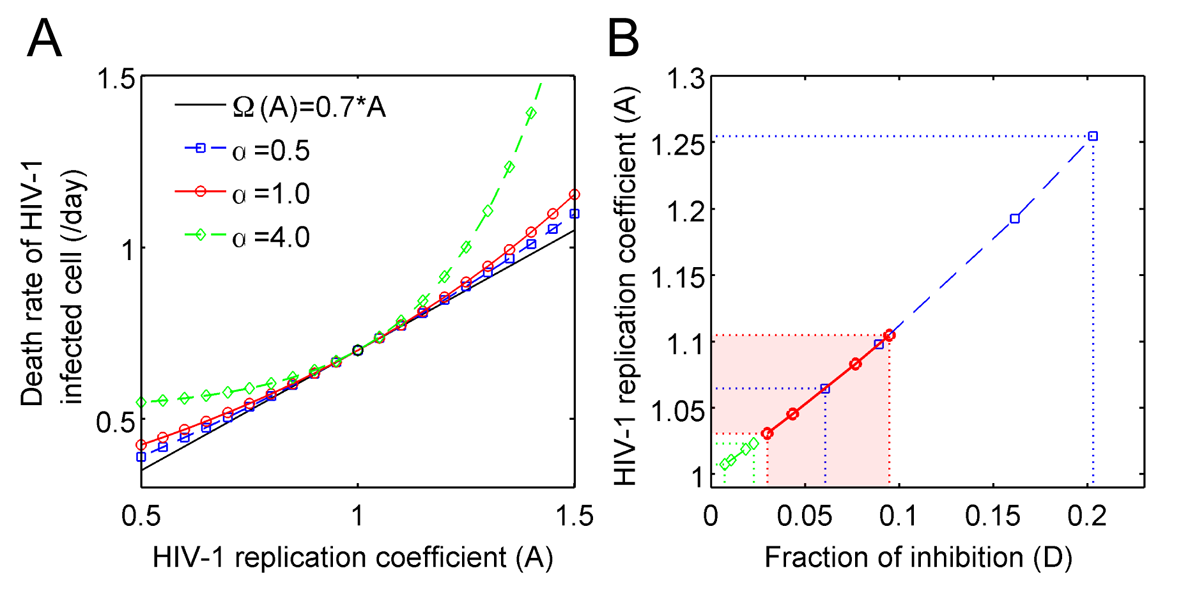

Supplement: Figure S1 — The optimal values of parameters A and D do not vary significantly with variation in the curvature of the cell death function, Ω() , or production ratio P . (A) Different shapes of the function Ω(A), which describes the dependence of T cell death rate on the production rate of HIV-1 gRNA (A), used for sensitivity analysis. The solid black line denotes a linear function. Other lines arise from the function with α values shown in the legend and the values of β, γ were chosen such that Ω(1) = 0.7. (B) The ranges of variation in optimal replication coefficient, A, and fraction of inhibition, D, under different assumptions of the cell death function Ω(A) shown in panel (A). Each line is color coded according to the assumptions on Ω(A) shown in panel (A). The optimal combinations of A and D are calculated with P varying from 5 to 30 (lines in the figure; circles correspond to values P = 5, 10, 20, 30, from left to right). The dashed lines show the range of variation in optimal A and D for each assumption on Ω (A); the red shaded area shows the range of variation for the functional form (α = 1) used in other simulations. (TIF) [file pcbi.1002744.s001.tif]

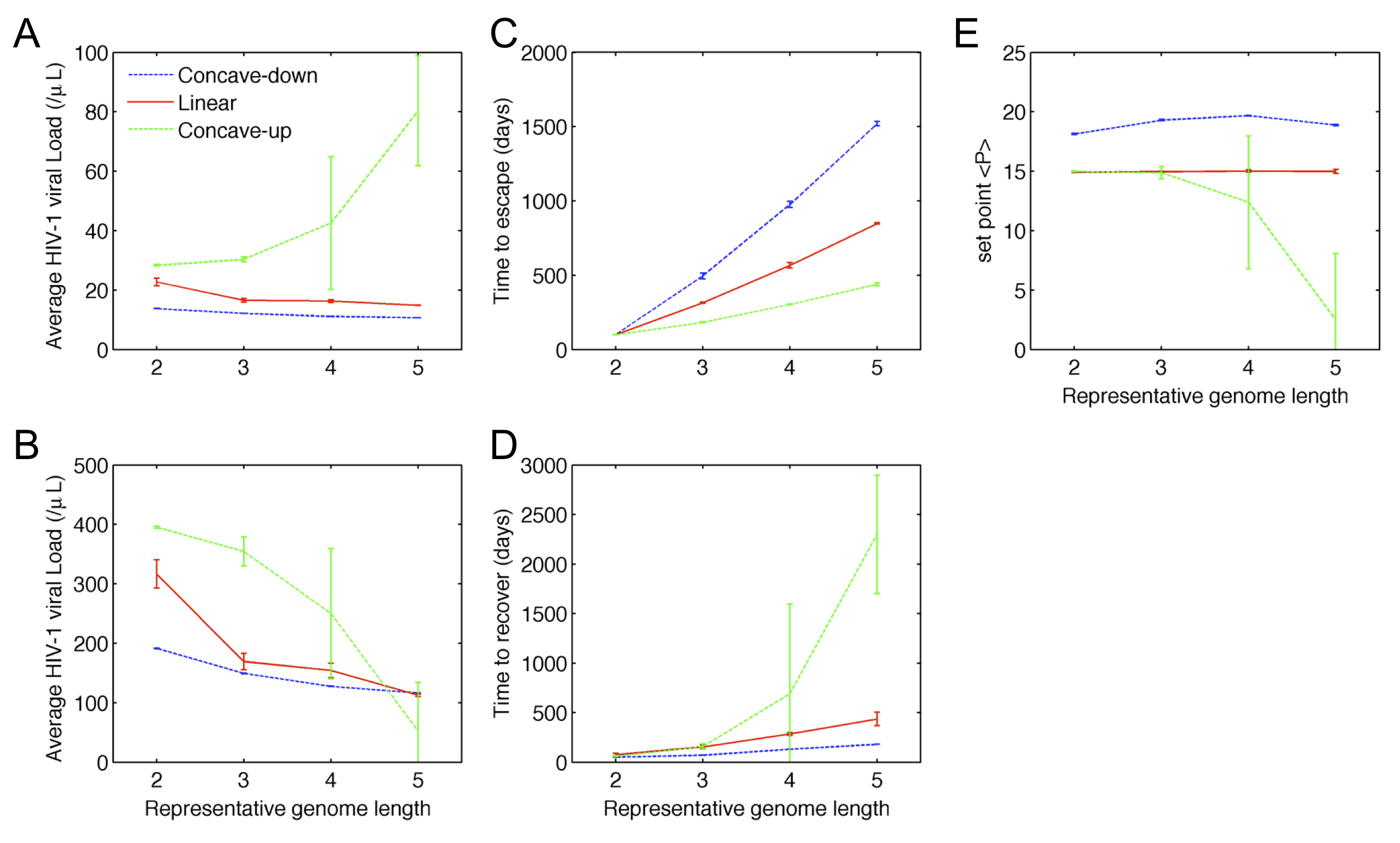

Supplement: Figure S2 — Co-evolutionary dynamics are qualitatively similar for models with alternative assumptions of genotype-phenotype mapping, although models assuming a concave-down function show better TIP performance. Five attributes are used to characterize the co-evolutionary dynamics of HIV-1 and TIP: the average HIV-1 and TIP viral loads (panels (A) and (B)) over the whole simulation (3000 days), the lengths of the PR phase and the ES phase (panels (C) and (D)) and the time-averaged mean value of P (panel (E)) during set-point phase. The values of D and A in these simulations are set to 0 and 1, respectively. 100 runs are performed for each data points. Data points and error bars correspond to the mean and standard deviation of 100 realizations of the model for those parameter values. Note that large standard deviations in data points for the concave-up function are due to TIP elimination events. (TIF) [file pcbi.1002744.s002.tif]

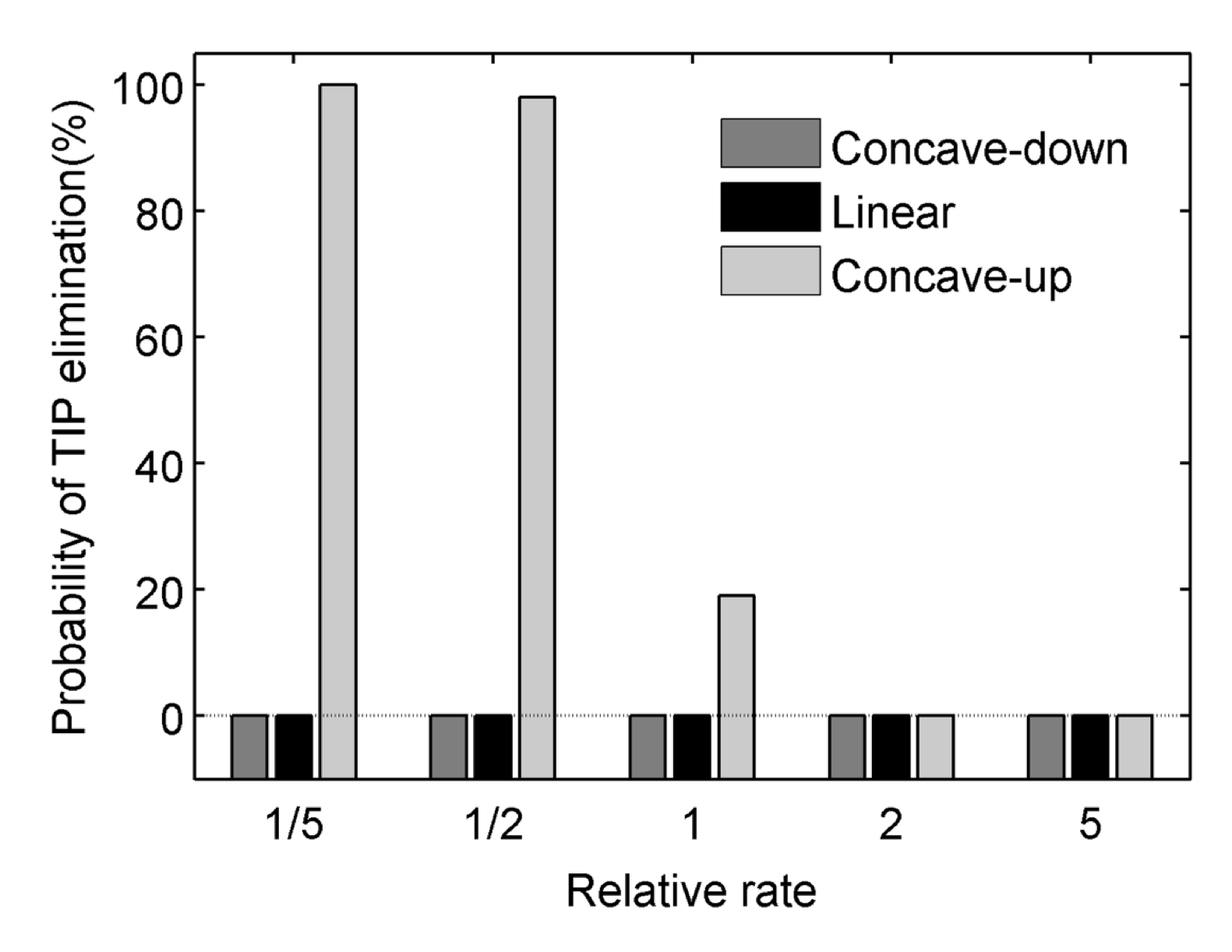

Supplement: Figure S3 — Effect of variations in the relative rate that TIP and HIV-1 mutations change the phenotypic parameter P . The probability of TIP elimination decreases as the relative rate of TIP evolution increases, when a concave-up genotype-phenotype mapping function is used. When the linear or concave-down mapping functions are used, the relative rate has no effect on TIP elimination. We represented the different rate at which mutations impacted the phenotypic parameter P by varying the mutation rate of the TIP genome in our genome-matching model. The ratios of TIP mutation rate over the HIV-1 mutation rate considered are 1/5, 1/2, 1, 2 and 5 in the simulations. For each ratio, 100 model runs were performed and the proportion of runs that exhibited TIP elimination is shown. Parameters A and D were kept constant at 1 and 0, respectively, in these simulations. (TIFF) [file pcbi.1002744.s003.tiff]

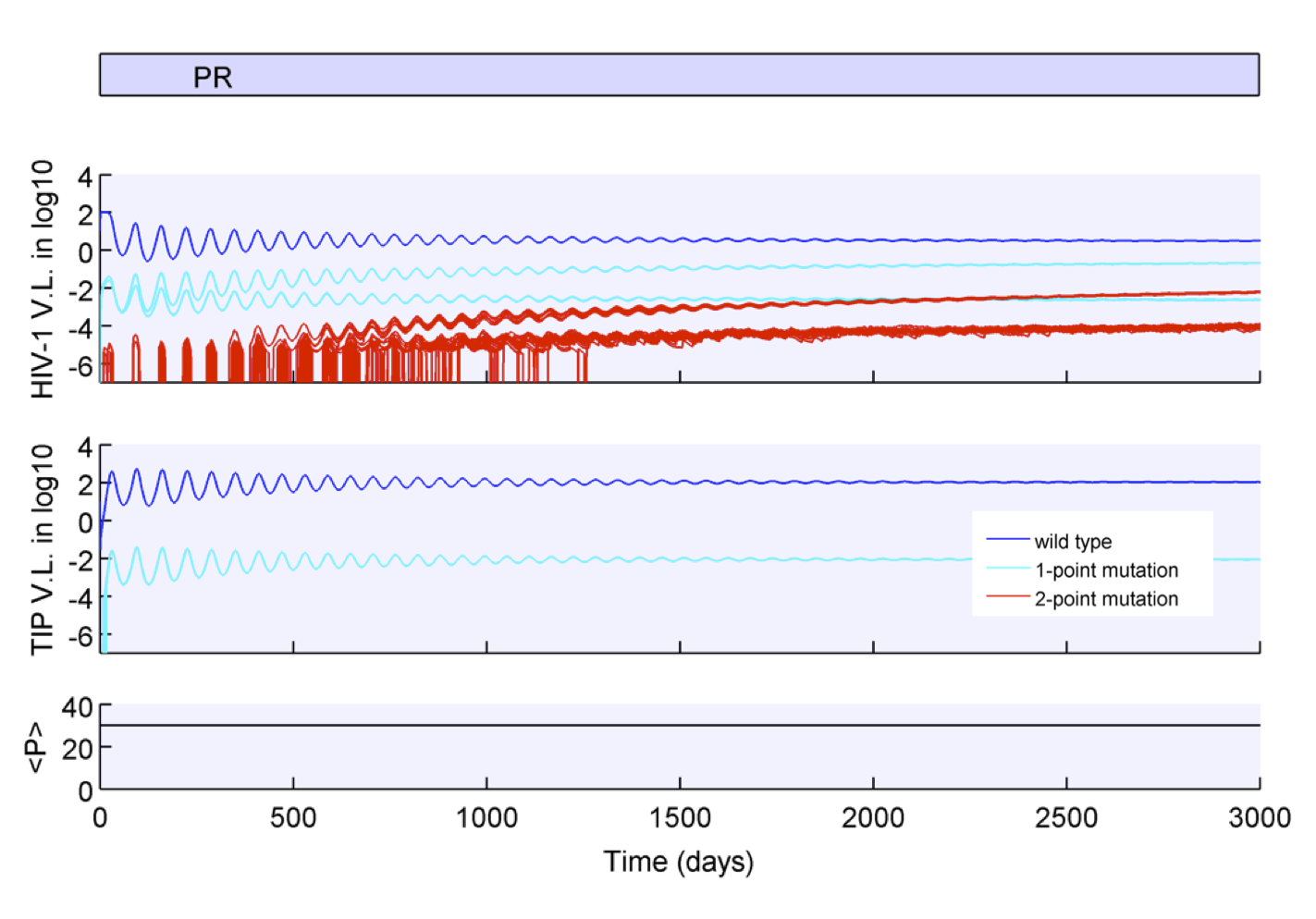

Supplement: Figure S4 — HIV-1 mutants are not selected when the cost of HIV-1 mutation is high. In this simulation, we assumed that the infectivity of all HIV-1 mutants is reduced by 30% compared to the wild-type infectivity. This assumption leads to reduced fitness for those HIV-1 mutants that are intermediate steps to the full-escape mutant as shown in Fig. 6. All other parameters are the same as in Fig. 5C. (TIFF) [file pcbi.1002744.s004.tiff]
