# Supplementary material for: Evolutionary Analysis of Human Immunodeficiency Virus Type 1 Therapies Based on Conditionally Replicating Vectors
Source: PLoS Comput Biol. 2012 Oct 25;8(10):e1002744. doi: 10.1371/journal.pcbi.1002744 (PMC3486895; doi:10.1371/journal.pcbi.1002744)
Supplement: Text S1 — Supplementary material. (PDF) [file pcbi.1002744.s005.pdf]

Supplementary Materials for

'Evolutionary Analysis of Human Immunodeficiency Virus 1 Therapies Based on Conditionally Replicating Vectors'

AUTHORS

Ruian Ke[1,†] and James O. Lloyd-Smith[1,2]

AFFILIATIONS

[1]Department of Ecology and Evolutionary Biology, University of California, Los Angeles, 610 Charles E. Young Dr. South, Los Angeles, CA 90095

[2]Fogarty International Center, National Institutes of Health, Bethesda, MD 20892

[†] To whom correspondence should be addressed. Email: ruian@ucla.edu

The intracellular model

In a T cell infected by only HIV-1 (denoted by x_i in Fig. 1), the production of HIV-1 genomes is modeled as:

$$\frac{dG_H^H}{dt} = A\cdot\theta - k_{pkg}\cdot {G_H^H}^2$$
$$\frac{dG_{HH}^H}{dt} = \frac{1}{2}\cdot k_{pkg}\cdot {G_H^H}^2 \qquad \text{(S1)}$$

The HIV-1 genomic RNAs (G_H^H) are produced at a rate of $A\theta$, where θ is the rate of genomic RNA (gRNA) replication for the wild-type HIV-1, and A models the replication rate of an HIV-1 strain relative to the wild-type, and we set A=1 for the wild-type. The HIV-1 genomic RNAs are dimerized (G_{HH}^H) at a rate k_{pkg} before being packaged into HIV-1 virions. Following Metzger *et al.*, we assume that the level of single-stranded HIV-1 gRNA reaches equilibrium quickly, and that packaging materials are present in excess [1]), so the dimerization of genomic RNAs is the limiting step in the process of viral particle formation. Thus, the rate of formation of new HIV-1 virions can be approximated as proportional to the rate of RNA dimerization. Substituting the solution for the equilibrium level of single-stranded genomes, we obtain:

$$\frac{dG_{HH}^H}{dt} = \frac{1}{2}\cdot A\cdot\theta \qquad \text{(S2)}$$

It also can be shown that the ratio of the rate of virion production in HIV-1 mutant infected cells over the rate in wild-type HIV-1 infected cells is A.

In a cell dually infected by HIV-1 and TIP (denoted by M_{ij} in Fig. 1), the dynamics of HIV-1 and TIP genomic RNAs are modeled as follows:

$$\frac{dG_H^M}{dt} = A \cdot (1-D) \cdot \theta - k_{pkg} \cdot G_H^{M\,2} - k_{pkg} \cdot G_H^M \cdot G_T^M$$
$$\frac{dG_T^M}{dt} = P \cdot A \cdot (1-D) \cdot \theta - k_{pkg} \cdot G_T^{M\,2} - k_{pkg} \cdot G_H^M \cdot G_T^M$$
$$\frac{dG_{HH}^M}{dt} = \frac{1}{2} \cdot k_{pkg} \cdot G_H^{M\,2}$$
$$\frac{dG_{HT}^M}{dt} = k_{pkg} \cdot G_H^M \cdot G_T^M \qquad \text{(S3)}$$
$$\frac{dG_{TT}^M}{dt} = \frac{1}{2} \cdot k_{pkg} \cdot G_T^{M\,2}$$

TIP gRNAs (G_T^M) are produced by co-opting the proteins required for transcription and molecular transport encoded by HIV-1 gRNAs (G_H^M). The dimerization of HIV-1 and TIP gRNAs results in three types of diploid genomes: homozygous HIV-1 (G_{HH}^M), homozygous TIP (G_{TT}^M) and heterozygous diploid genomes (G_{HT}^M). We assume that genome assortment is random, i.e. the single-stranded genomes partition binomially into diploid genomes as observed in recent experiments [1].

Once again, we assume that levels of single stranded genomic RNAs reach equilibrium quickly in dually infected cells, and so the rates of change of diploid genome production become constants. By solving the first two equations in ordinary differential equations (ODEs) (S3) at equilibrium and following the same argument as for singly infected cells, we derive the approximation to the rate of homozygous HIV-1 and TIP production rate as:

$$\frac{dG_{HH}^M}{dt} = \frac{1}{2} \cdot \frac{(1-D)}{(1+P)} \cdot \theta, \qquad \frac{dG_{TT}^M}{dt} = \frac{1}{2} \cdot \frac{P^2 \cdot (1-D)}{(1+P)} \cdot \theta \quad \text{(S4)}$$

The total numbers of different types of virions produced by a cell before it dies are proportional to the rates of virion production, so the relative quantities of different virions are determined by ratios of these rates. Thus, we calculate ψ and ρ by substituting equations (S2) and (S4) into the last three equations in ODEs (S3), and we get [2]:

$$\psi = \frac{dG_{HH}^M / dt}{dG_{HH}^H / dt} = \frac{1-D}{1+P} \qquad \text{(S5)}$$

and

$$\rho = \frac{dG_{TT}^M / dt}{dG_{HH}^M / dt} = P^2 \qquad \text{(S6)}$$

The HIV-1 mutant model

The HIV-1 mutant model is a simplified version of the General Model. It only considers the wild-type HIV-1, the wild-type TIP and a mutant HIV-1 strain. The full ODEs are as follows:

$$
\begin{aligned}
\frac{dU}{dt} &= \lambda - k \cdot x_0 \cdot U - k \cdot x_1 \cdot U - k \cdot y_0 \cdot U - d \cdot U \\
\frac{dH_0}{dt} &= k \cdot x_0 \cdot U - \Omega(A_0) \cdot H_0 \\
\frac{dH_1}{dt} &= k \cdot x_1 \cdot U - \Omega(A_1) \cdot H_1 \\
\frac{dT_0}{dt} &= k \cdot y_0 \cdot U - k \cdot x_0 \cdot T_0 - k \cdot x_1 \cdot T_0 - d \cdot T_0 \\
\frac{dM_{00}}{dt} &= k \cdot x_0 \cdot T_0 - \Omega(A_0 \cdot (1 - D_{00})) \cdot M_{00} \\
\frac{dM_{10}}{dt} &= k \cdot x_1 \cdot T_0 - \Omega(A_1 \cdot (1 - D_{10})) \cdot M_{10} \\
\frac{dx_0}{dt} &= \pi \cdot A_0 \cdot H_0 + \psi_{00} \cdot \pi \cdot A_0 \cdot M_{00} - c \cdot x_0 \\
\frac{dx_1}{dt} &= \pi \cdot A_1 \cdot H_1 + \psi_{10} \cdot \pi \cdot A_1 \cdot M_{10} - c \cdot x_1 \\
\frac{dy_0}{dt} &= \rho_{00} \cdot \psi_{00} \cdot \pi \cdot A_0 \cdot M_{00} + \rho_{10} \cdot \psi_{10} \cdot \pi \cdot A_1 \cdot M_{10} - c \cdot y_0
\end{aligned}
\qquad \text{(S7)}
$$

The TIP mutant model

Similarly, the TIP mutant model is a simplified version of the General Model, which only considers wild-type HIV-1, wild-type TIP and a mutant TIP strain. The full ODEs are as follows:

$$\frac{dU}{dt} = \lambda - d\cdot U - k\cdot x_0\cdot U - k\cdot y_0\cdot U - k\cdot y_1\cdot U$$
$$\frac{dH_0}{dt} = k\cdot x_0\cdot U - \Omega(A_0)\cdot H_0$$
$$\frac{dT_0}{dt} = k\cdot y_0\cdot U - d\cdot T_0 - k\cdot x_0\cdot T_0$$
$$\frac{dT_1}{dt} = k\cdot y_1\cdot U - d\cdot T_1 - k\cdot x_0\cdot T_1$$
$$\frac{dM_{00}}{dt} = k\cdot x_0\cdot T_0 - \Omega(A_0\cdot (1 - D_{00}))\cdot M_{00}$$
$$\frac{dM_{01}}{dt} = k\cdot x_0\cdot T_1 - \Omega(A_0\cdot (1 - D_{01}))\cdot M_{01}$$
$$\frac{dx_0}{dt} = \pi\cdot A_0\cdot H_0 - c\cdot x_0 + \psi_{00}\cdot \pi\cdot A_0\cdot M_{00} + \psi_{01}\cdot \pi\cdot A_0\cdot M_{01} \quad \text{(S8)}$$
$$\frac{dy_0}{dt} = \rho_{00}\cdot \psi_{00}\cdot \pi\cdot A_0\cdot M_{00} - c\cdot y_0$$
$$\frac{dy_1}{dt} = \rho_{01}\cdot \psi_{01}\cdot \pi\cdot A_0\cdot M_{01} - c\cdot y_1$$

Mutations in DIS sequence

The Dimerization Initiation Signal (DIS) is a 6-nucleotide palindromic sequence on stem-loop 1 (SL1) of the HIV-1 genome [3]. It has been shown that DIS plays a critical role in the pairing between HIV-1 genomic RNAs [4,5,6]. The probability of pairing between different gRNAs is directly dependent on the extent of complementarity (in terms of Watson-Crick base pairs) between the DIS sequences on the two gRNA copies [1]. The DIS sequence is highly conserved in the HIV-1 genome, being GCGCGC. The DIS sequence in TIP is designed to be GCGCGC, such that HIV-1 gRNAs are efficiently paired with TIP gRNAs, resulting in a maximal level of heterodimer formation in dually infected cells. Because the heterozygote particle is not viable, a large proportion of HIV-1 gRNAs are diverted from forming HIV-1 virions in this way. Since heterodimer formation wastes resources for both HIV-1 and TIP, it can be envisaged that both HIV-1 and TIP evolution might favor decreased heterodimer formation, which would increase the proportion of HIV-1 and TIP homodimers formed, thereby increasing the amounts of HIV-1 and TIP virions produced in dually infected cells. This is clearly an important consideration in designing TIPs, since DIS mutations that reduce heterodimer formation would undermine the long-term efficacy of TIP. However, experiments have shown that mutations in the DIS sequence lead to marked reductions in viral replication and infectivity, which are directly related to viral fitness [4,5,6]. It is not clear whether these costs would outweigh the benefits to HIV-1 or TIP, and hence whether

mutations in the DIS sequence should be expected to occur.

To model the impact of DIS mutation on the distribution of dimers, we modified the intracellular model by adding in another dimensionless parameter *W*, which is the relative rate of heterodimer formation compared to homodimer formation. Mutations in the DIS could change the formation rate of all three types of dimers independently, but to focus the analysis on the concern raised above, we assumed that DIS mutations lead to changes only in the relative rate of heterodimer formation, i.e. the parameter *W*. Since we have shown in the main text that the values of parameters *A* and *D* stay at 1 and 0, respectively, in the long-term co-evolutionary dynamics, we set *A*=1 and *D*=0 in the analyses below.

The ODEs for the modified intracellular model are:

$$\begin{aligned}
\frac{dG_H}{dt} &= \theta - k_{pkg} \cdot G_H^2 - W \cdot k_{pkg} \cdot G_H \cdot G_T \\
\frac{dG_T}{dt} &= P \cdot \theta - k_{pkg} \cdot G_T^2 - W \cdot k_{pkg} \cdot G_H \cdot G_T \\
\frac{dG_{HH}^M}{dt} &= \frac{1}{2} \cdot k_{pkg} \cdot G_H^2 \\
\frac{dG_{HT}^M}{dt} &= W \cdot k_{pkg} \cdot G_H^2 \\
\frac{dG_{TT}^M}{dt} &= \frac{1}{2} \cdot k_{pkg} \cdot G_T^2
\end{aligned} \qquad \text{(S9)}$$

The values of ψ and ρ for the intracellular model above can then be derived from equations (S9):

$$\psi = \frac{P \cdot W^2 + 2 - W^2 - \sqrt{W^2 \cdot (P^2 \cdot W^2 + 4 \cdot P - 2 \cdot P \cdot W^2 + W^2)}}{2 \cdot (1 - W^2)} \qquad \text{(S10)}$$

and

$$\rho = \frac{(-P \cdot W - W + \sqrt{P^2 \cdot W^2 + 4 \cdot P - 2 \cdot P \cdot W^2 + W^2})^2}{(P \cdot W^2 + 2 - W^2 - \sqrt{W^2 \cdot (P^2 \cdot W^2 + 4 \cdot P - 2 \cdot P \cdot W^2 + W^2)})^2} \qquad \text{(S11)}$$

To examine the fitness of the HIV-1 and TIP mutants with mutated DIS, we performed two sets of invasibility analyses (using the 3-strain 'mutant' models) for either a mutant HIV-1 strain or a mutant TIP strain that has an altered DIS sequence. In the first analysis, to check our intuition about the evolutionary pressure on the dimer distribution, we assumed that mutations in the DIS sequence do

not reduce viral replication or infectivity, i.e. there is no cost associated with DIS mutation for either HIV-1 or TIP. In the second analysis, we considered the more realistic scenario based on previously published experimental data, in which DIS mutations reduce viral infectivity.

Analysis I: no fitness cost for DIS mutations

We first consider the scenario that mutations in the DIS do not change the infectivity of HIV-1 or TIP in a dually infected host. In this model, the sole effect of mutations in the DIS is to change the distribution of heterodimers in dually infected cells. This leads to changes in production rates of HIV-1 and TIP particles at the within-host level, i.e. the parameters ψ and ρ, and we assume that other parameter values in the within-host model are unaffected. To test the invasibility of DIS mutated strains, we substitute the expressions of ψ and ρ in Eqns. (S10) and (S11) for the mutant strain into the fitness expressions for HIV-1 mutants ($R_{eff,H}$ in Eqn.(5) in the main text) and TIP mutants ($R_{eff,T}$ in Eqn.(6)) in the two 3-strain mutant models.

We plotted the impact of changes in parameters *W* and *P* on the fitnesses of HIV-1 and TIP (Fig. S5). As we anticipated, HIV-1 and TIP mutants with lower values of *W* always have higher fitness, for any value of *P*. This agrees with our intuition that a lower rate of heterodimer formation results in higher production rates of both HIV-1 and TIP virions, and thus mutants with lower rates of heterodimer formation will be selected if mutating the DIS does not cause significant reductions in viral packaging and infectivity.

While our analysis shows that HIV-1 and TIP are both prone to evolve towards lower *W*, the impacts of reducing *W* on HIV-1 and TIP fitness are markedly different. The fitness of HIV-1 rises much more sharply when *W* is low, compared to the fitness increase for TIP. This is because TIP gRNA is produced at a much higher rate than HIV-1 gRNA, so the proportion of available gRNAs that ends up in heterodimers is much higher for HIV-1 than for TIP. This leads to fewer homodimers of HIV-1 genomes, and hence fewer virions produced by the cell, i.e. the fitness of HIV-1 is more affected by changes in the rate of heterodimer formation than that of TIP.

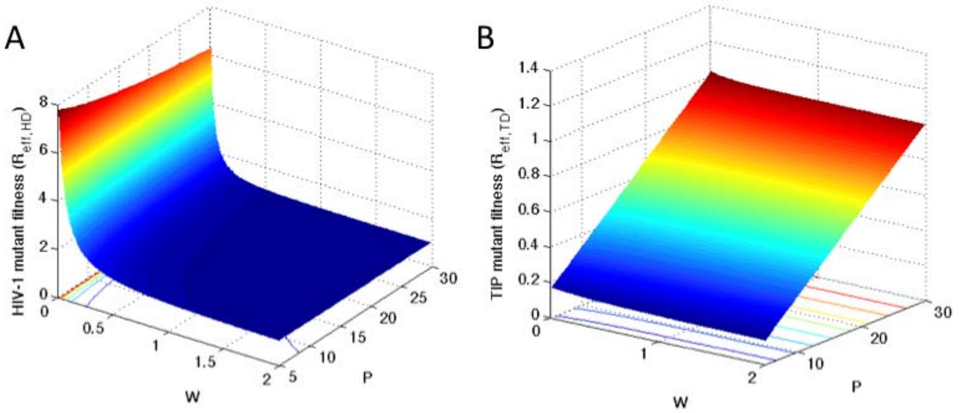

Figure S5. Mutants with lower rates of heterodimer formation (*W*) have selective advantages over the wild-type trains. The fitness of HIV-1 mutants (A) and TIP mutants (B) corresponding to different values of *W* and *P* in dually infected cells. Note that in panel (B), the maximal fitness for any given value of *P* is always obtained when *W*=0.

Analysis II: DIS mutations reduce viral infectivity

In the second analysis, we assumed that mutations in DIS reduce viral infectivity in addition to their impact on the rate of heterodimer formation. In the '3-strain' models for within-host dynamics, we modeled the mutant infectivity with a new parameter k_1 to differentiate it from the wild-type infectivity (k). To investigate the trade-off between the lower rate of heterodimer formation and the reduction in infectivity, we assumed that mutation in the DIS leads to a 25% reduction in infectivity, and that the fraction of heterodimer is reduced from 50% to 20% in dually infected cells that are infected with the mutant strain. These assumptions are based on the following experimental observations:

1. Chen et al. measured the distribution of different diploid genomes in cells co-infected by a series of combinations of two HIV-1 strains with different DIS sequences [1]. The lowest proportion of heterodimer formation obtained from cells dually infected with two HIV-1 strains was 20% of the total population, which translates to *W*=0.25 in our model. The DIS sequences of these two HIV-1 strains are GGGGGG and CCCCCC. Each sequence is complementary to the other, but not to itself.
2. Several studies have measured the impact of mutations in the DIS on viral packing and infectivity [4,5,6]. In general, it is observed that strains with DIS mutations have more than 25% reduction in replication efficiency and infectivity. In the model, we assume $k_1=0.75*k_0$.

Thus we have interpreted the available data to make the most conservative parameter estimates (i.e. the scenario most favorable for the DIS mutants), in that the mutant strains are assumed to have the

lowest heterodimer formation and highest infectivity among the experimentally observed DIS mutants. These assumptions correspond to parameter values of W=0.25 and k_1=0.75*k_0. As in the first analysis, we set A=1 and D=0, and we assume that the value of P is the same in all dually infected cells.

The detailed within-host ODEs are omitted here, but are directly analogous to the '3-strain' mutant models. The effective reproduction number for the DIS-mutated HIV-1 mutant ($R_{eff,HD}$) can be derived:

$$R_{eff,HD} = \frac{k_1 \cdot \pi \cdot \Omega(1) \cdot U^*}{(c \cdot \Omega(1) - k_1 \cdot \pi \cdot \psi_{10} \cdot T_0^*) \cdot \Omega(1)} \qquad \text{(S12)}$$

where U^* and T_0^* are the equilibrium levels of uninfected T cells and T cells infected by the wild-type TIP in the absence of HIV-1 mutants, respectively. The parameter ψ_{10} is the value of ψ in cells dually infected with the mutant HIV-1 and the wild-type TIP, and its value is given by Eqn. S10.

Similarly, the effective reproduction number for the DIS-mutated TIP mutant ($R_{eff,TD}$) can be derived:

$$R_{eff,TD} = \frac{k_1 \cdot \rho_{01} \cdot \psi_{01}}{k \cdot \rho_{00} \cdot \psi_{00}} \qquad \text{(S13)}$$

where ψ_{00} and ρ_{00} are the parameter values in cells dually infected with the wild-type HIV-1 and TIP. ψ_{01} and ρ_{01} are the parameter values in cells dually infected with the wild-type HIV-1 and the mutant TIP, and they are given in Eqn. S10 and S11.

We calculated the values of $R_{eff,HD}$ and $R_{eff,TD}$ in the corresponding two models with different values of parameter P in dually infected cells. For the HIV-1 mutant, $R_{eff,HD}$ is always less than 1 for wide ranges of possible values of P (Fig. S6A), i.e. the mutant HIV-1 is not able to invade over wild-type HIV-1. For the TIP mutant, again we found that $R_{eff,TD}$ is also always less than 1 in the range of P values that allow mutant TIP to invade (Fig. S6B), so the mutant TIP is not able to invade the wild-type TIP.

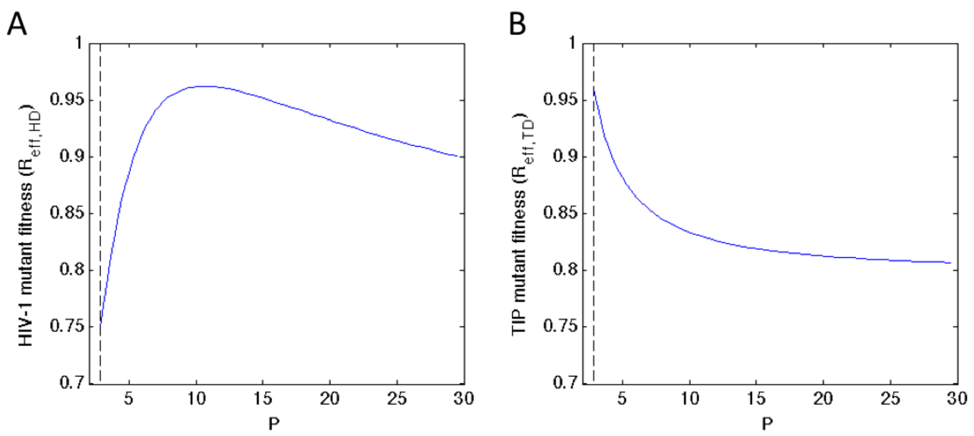

Figure S6. HIV-1 and TIP strains with mutations in their DIS sequence are less fit than strains with wild-type DIS. **(A)** Variations of HIV-1 mutant fitness for different values of P. **(B)** Variations of TIP mutant fitness for different values of P. The peak value of $R_{eff,HD}$ and $R_{eff,TD}$ are 0.962 and 0.960, respectively. The dashed line in panel (B) denotes the invasion threshold for TIP ($P_{threshold}$).

Conclusion

To summarize, our analyses show that mutations in the DIS regions of the HIV-1 or TIP genomes can provide a fitness benefit to both populations by increasing the proportion of viable homodimers formed. If DIS mutations do not impose costs by affecting viral packaging or infectivity, then selection will favor a lower rate of heterodimer formation for both HIV-1 and TIP. However, when we incorporate dimerization patterns and reduction in viral infectivity as reported in the literature, we see that the fitness costs outweigh the benefits and DIS mutations appear unlikely to be favorable. This analysis indicates that the advantageous effect of high rates of heterodimer formation is likely to be evolutionarily robust, but we note that the safety margin is small ($R_{eff,HD}$ and $R_{eff,TD}$ are both close to 1) and that we have made numerous other assumptions in our analysis. While we have interpreted available data conservatively, these findings highlight the fact that DIS mutations are a potential vulnerability of the TIP strategy, and ensuring robustness of heterodimer formation – or incorporating other robust mechanisms for TIP to outcompete HIV-1 within a cell – should be a focus for gene therapy design.

In our intracellular model, we have assumed that the only limiting factor for production of both HIV-1 and TIP is the dimerization of gRNAs, and that the materials required for viral replication and packaging are abundant. As a consequence, the model depicts resource competition between HIV-1 and TIP as arising only from heterodimer formation, i.e. the fitness cost to HIV-1 in dually infected cells is assumed to be the pairing between HIV-1 and TIP gRNAs. This is why lower rates of

heterodimer formation are favored by both HIV-1 and TIP. In the extreme case, when no heterodimer is formed (W=0), and there is no direct inhibition (D=0), this model assumes that the presence of TIP does not induce any fitness cost to HIV-1. However, for TIP and for conditionally replicating vectors (CRVs) in general, competition for resources can arise from other stages of the HIV-1 life-cycle, such as for proteins needed in viral replication and packaging. These aspects are not considered here, which is another sense in which our analysis is a conservative estimate for the efficacy of TIP. Explicit modeling analysis would be needed to understand the evolutionary implications of other sources of competition between HIV-1 and CRVs for any specific gene therapy strategy.

Sensitivity analysis - rate of superinfection (k')

Because the TIP can replicate only in the presence of HIV-1, the frequency of dually infected cells will be a key determinant of the viability of TIP therapy. In the model analyzed in the main text, we have assumed that dually infected cells are derived only through the superinfection of TIP infected cells by HIV-1 and that the rate of superinfection of TIP-infected T cells by HIV-1 is the same as the infection rate of naïve cells, following the work of Weinberger *et al.* [7]. Here, we first test whether the superinfection rate assumed in our model is consistent with a recent study showing that the percentages of multiply HIV-1-infected $CD4^+$ T cells in peripheral blood *in vivo* are 2.6% and 7.0% (upper bounds of 95% confidence interval, 19% and 29%) for acute and chronic infection, respectively [8]. We then evaluate the impact of including an additional pathway of superinfection, where HIV-1 infected cells can be superinfected by TIP, and test the sensitivity of our main conclusions to variations in the superinfection rate.

Fraction of cells superinfected

We have assumed that the superinfection of HIV-1 infected cells by TIP does not occur, and our model only keeps track of the dually infected cells that are infected by one HIV-1 and one TIP virion. Therefore, the fraction of dually infected cells in our model for HIV-1 and TIP will be an underestimate of the true number of dually infected cells (which would include cells dually infected with two TIP virions, for instance). To test the consistency of the superinfection rate assumed in our model with the experimental data, we consider a model without TIP but including superinfection of T cells with two HIV-1 virions, similar with the experimental setting in the study of Josefsson *et al.* [8]. The fraction of dually infected cells predicted by this model, using the superinfection rate assumed in our HIV-1/TIP model, then can be compared with the experimental estimates.

The ODEs for the model can be written as:

$$\frac{dU}{dt} = \lambda - k \cdot x \cdot U - d \cdot U$$
$$\frac{dH}{dt} = k \cdot x \cdot U - \delta \cdot H - k' \cdot x \cdot H \qquad \text{(S14)}$$
$$\frac{dY}{dt} = k' \cdot x \cdot H - \delta \cdot Y$$
$$\frac{dx}{dt} = \pi \cdot (H + Y) - c \cdot x$$

where *Y* is the T cells superinfected by two HIV-1 virions, *k′* is the rate of superinfection, δ is the death rate of HIV-1 infected cells, and here we assume $\delta = \Omega(1)$. Other state variables and parameter values follow the definitions in our main model Eqn. (S7).

Following the approach of Althaus and De Boer [9], we calculate the fraction *f* of superinfected cells among all infected cells in the system at equilibrium as a function of the superinfection rate *k′*:

$$f(k') = \frac{\lambda \cdot k \cdot \pi - \delta \cdot c \cdot d}{\lambda \cdot k \cdot \pi - \delta \cdot c \cdot d + \delta^2 \cdot c \cdot k/k'} \qquad \text{(S15)}$$

We have set the superinfection rate for HIV-1 in our model equal to the rate of infection of naïve cells, i.e. *k*=*k′*. Substituting all the parameter values used in our model, we get *f*(*k′*)=0.026. That is, the fraction of dually infected cells would be 2.6% under the assumptions of our model, which is very close to the fraction reported by Josefsson *et al.* [8] for acute infection. Therefore, the superinfection rate assumed in the model analyzed in the main text is consistent with the recent measurements of the fraction of dually infected cells [8].

The fraction of cells dually infected by HIV-1 and TIP in our model simulations is much lower than the predicted fraction from Eqn. (S15) (see Fig. S7 below). This is because the great majority of infected cells are infected by TIP, and TIP virions far outnumber HIV-1 virions. Therefore cells dually infected by two TIPs, which are not considered in our study, constitute the majority of all dually infected cells. We do not expect those cells dually infected by TIPs to have a substantial impact on the co-evolutionary dynamics, since only a negligible fraction of them would be further infected by HIV-1 given the current estimate of the frequency of dual infections.

Invasion threshold

To explore the impact of including superinfection of HIV-1 infected cells by TIPs, we modified our

model to include this infection route. Including this infection route does not change the direction of selection pressures on HIV-1 and TIP along the three parameters *P, D* and *A*, since adding this superinfection route affects all variants in the same way. However, the higher rate of superinfection leads to higher level of dually infected cells, and therefore it will lower the minimum value of *P* required for TIP invasion, i.e. the invasion threshold $P_{\text{threshold}}$ for TIP. We thus derive the reproductive value $R_{0,\text{T}}$ for the model incorporating this additional superinfection route, in order to draw conclusions about the value of $P_{\text{threshold}}$.

For a model considering only the wild-type HIV-1 and TIP, the ODEs can be written as following:

$$
\begin{aligned}
\frac{dU}{dt} &= \lambda - k \cdot x \cdot U - k \cdot y \cdot U - d \cdot U \\
\frac{dH}{dt} &= k \cdot x \cdot U - \Omega(A) \cdot H - k' \cdot y \cdot H \\
\frac{dT}{dt} &= k \cdot y \cdot U - d \cdot T - k' \cdot x \cdot T \\
\frac{dM}{dt} &= k' \cdot y \cdot H + k' \cdot x \cdot T - \Omega(A(1-D)) \cdot M \\
\frac{dx}{dt} &= \pi \cdot A \cdot H + \pi \cdot A \cdot \psi \cdot M - c \cdot x \\
\frac{dy}{dt} &= \pi \cdot A \cdot \rho \cdot \psi \cdot M - c \cdot y
\end{aligned}
\qquad \text{(S16)}
$$

The state variables and parameters follow the same meaning as in the main text.

The expression for the reproductive number ($R_{0,\text{T}}$) of TIP when it is introduced in a system with HIV-1 at non-zero equilibrium can be derived:

$$
R_{0,T} = \frac{k \cdot k' \cdot \pi \cdot A \cdot \rho \cdot \psi \cdot x' \cdot U'}{\left(c \cdot \Omega\big(A \cdot (1-D)\big) - k' \cdot \pi \cdot A \cdot \rho \cdot \psi \cdot I'\right) \cdot (k' \cdot x' + d)} \qquad \text{(S17)}
$$

where *x'*, *I'* and *U'* are the equilibrium levels of HIV-1 virions, HIV-1-infected cells and uninfected T cells, respectively, in the absence of TIP.

By setting *k=k'*, we can compare Eqn. (S15) to the expression for $R_{0,\text{T}}$ for our main model (Eqn. (4) in the Methods section in the main text), and we find that including this superinfection route increases the value of $R_{0,\text{T}}$. The fitness of TIP is increased, which is intuitive because more cells will be coinfected, so TIP has more opportunities to spread. Therefore, a lower value of $P_{\text{threshold}}$ is needed for TIP to invade the HIV-1 population. Given the role that the value of $P_{\text{threshold}}$ plays in the persistence of TIP in the long term co-evolutionary dynamics, as analyzed in the main text, this result

suggests that the persistence of TIPs would be enhanced if TIPs are able to infect HIV-1-infected cells. Furthermore, $R_{0,T}$ increases monotonically with *k′* irrespective of the values of *P*, *D* and *A*, suggesting that superinfection rates higher than the rate investigated in our model will uniformly favor TIP persistence.

Temporal co-evolutionary dynamics is robust to variations in the superinfection rate k′

To investigate how the temporal evolutionary dynamics change with respect to different rates of superinfection, we extended the full multi-strain model by allowing for superinfection of HIV-1-infected cells by TIP. We then performed simulations with the rate of superinfection *k′* to be 1, 3, 5 and 7 fold higher than the value of *k*. This corresponds to 2.6%, 7.4%, 11.8% and 15.8% of all infected cells being dually infected according to Eqn. (S17).

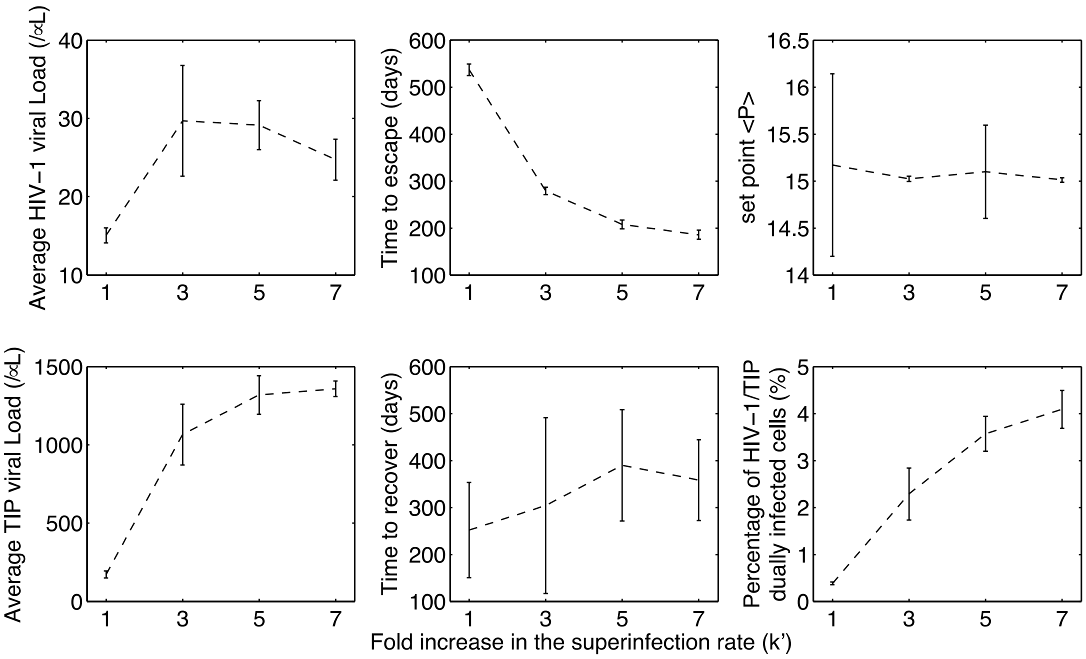

Figure S7. The three-phase pattern of HIV-1 and CRVs co-evolutionary dynamics is robust to changes in the rate of superinfection (*k′*). Note that setting the rate of superinfection *k′* to be 1, 3, 5 and 7 fold of the value of *k* corresponds to 2.6%, 7.4%, 11.8% and 15.8% of all infected cells being dually infected according to Eqn. (S17).

We find that the three-phase pattern of HIV-1 and CRVs co-evolutionary dynamics is robust to variation in the superinfection rate (Fig. S7). The percentage of cells dually infected by HIV-1 and TIP increases as the superinfection rate increases. Higher superinfection rates enhance the inhibition of wild-type HIV-1 production by wild-type TIP, and hence increase the selection pressure on HIV-1 to

escape. As a result, the full mutant HIV-1 strains are generated earlier in models with higher superinfection rates (note the rapid decrease in the 'time to escape' with increases in *k'* in Fig. S7). The time for TIP to recover increases slightly for higher superinfection rates, owing to greater instability in strain dynamics. Together, these changes in timescale lead to a slightly increased average HIV-1 viral load over 3000 days. However, the HIV-1 viral loads in the set-point phase remain the same for different superinfection rates, as indicated by the constant set-point value of *P* shown in Fig. S7.

Because the three-phase pattern of the co-evolutionary dynamics is robust to changes in the superinfection rate and frequency of dual infection, our results should apply to a wide range of situations including acute and chronic HIV-1 infection.

SUPPLEMENTARY REFERENCES

1. Chen J, Nikolaitchik O, Singh J, Wright A, Bencsics CE, et al. (2009) High efficiency of HIV-1 genomic RNA packaging and heterozygote formation revealed by single virion analysis. Proc Natl Acad Sci U S A 106: 13535-13540.

2. Metzger VT, Lloyd-Smith JO, Weinberger LS (2011) Autonomous targeting of infectious superspreaders using engineered transmissible therapies. PLoS Comput Biol 7: e1002015.

3. Skripkin E, Paillart JC, Marquet R, Ehresmann B, Ehresmann C (1994) Identification of the primary site of the human immunodeficiency virus type 1 RNA dimerization in vitro. Proc Natl Acad Sci U S A 91: 4945-4949.

4. Moore MD, Fu W, Nikolaitchik O, Chen J, Ptak RG, et al. (2007) Dimer initiation signal of human immunodeficiency virus type 1: its role in partner selection during RNA copackaging and its effects on recombination. J Virol 81: 4002-4011.

5. Clever JL, Parslow TG (1997) Mutant human immunodeficiency virus type 1 genomes with defects in RNA dimerization or encapsidation. J Virol 71: 3407-3414.

6. Berkhout B, van Wamel JL (1996) Role of the DIS hairpin in replication of human immunodeficiency virus type 1. J Virol 70: 6723-6732.

7. Weinberger LS, Schaffer DV, Arkin AP (2003) Theoretical design of a gene therapy to prevent AIDS but not human immunodeficiency virus type 1 infection. J Virol 77: 10028-10036.

8. Josefsson L, King MS, Makitalo B, Brannstrom J, Shao W, et al. (2011) Majority of CD4+ T cells from peripheral blood of HIV-1-infected individuals contain only one HIV DNA molecule. Proc Natl Acad Sci U S A 108: 11199-11204.

9. Althaus CL, De Boer RJ (2012) Impaired immune evasion in HIV through intracellular delays and multiple infection of cells. Proc Biol Sci.
